# Coordination Geometry Tuning in a Single‐Atom Nanozyme to Mimic Metalloenzymes with Nonplanar Active Site

**DOI:** 10.1002/advs.202505733

**Published:** 2025-08-09

**Authors:** Hyesung Lee, Changjoon Keum, Choah Kwon, Sangtae Kim, Youngdo Jeong, Sang‐Yup Lee

**Affiliations:** ^1^ Department of Chemical and Biomolecular Engineering Yonsei University Seoul 03722 Republic of Korea; ^2^ Center for Advanced Biomolecular Recognition Biomedical Research Division Korea Institute of Science and Technology (KIST) Seoul 02792 Republic of Korea; ^3^ Department of Nuclear Engineering and Department of Materials Science and Engineering Hanyang University Seoul 04763 Republic of Korea; ^4^ Department of HY‐KIST Bio‐convergence Hanyang University Seoul 04763 Republic of Korea

**Keywords:** carbonic anhydrase, CO_2_, coordination geometry, hydrolysis, single‐atom nanozyme

## Abstract

Single‐atom nanozymes (SAzymes) have been developed to mimic metalloenzymes by modulating their coordination environment. However, the impact of the coordination geometry at the metal center on the catalytic activity of the SAzymes, particularly those with nonplanar active site configurations, has been minimally explored. Here, the effects of the geometric configuration of the Zn–N_4_ active sites on the catalytic activity of the carbonic anhydrase (CA)‐mimetic SAzymes are reported. CA‐mimetic SAzymes with Zn–N_4_ active sites in tetrahedral, distorted tetrahedral, and square‐planar configurations are prepared by simple temperature‐controlled carbonization of ZIF‐8. The SAzyme with the distorted tetrahedral Zn–N_4_ configuration displays superior catalytic CO_2_ hydration activity, attributed to increased Lewis acidity. Density functional theory (DFT) calculations reveal a pronounced Zn–OH affinity and localized electron accumulation at the distorted tetrahedral Zn site. These results demonstrate the critical role of precise geometric tuning in enhancing SAzyme efficacy and can be used to facilitate potential avenues for optimizing metal active centers in enzyme mimetics.

## Graphical abstract

Correlation between coordination geometry and catalytic activity of carbonic anhydrase‐mimetic single‐atom Zn–N_4_ active sites is revealed. Among tetrahedral, distorted tetrahedral, and square‐planar Zn–N_4_ geometric configurations, the distorted tetrahedral Zn–N_4_ exhibits superior CO_2_ hydrolysis performance. This is attributed to increased Lewis acidity, Zn–OH affinity, and localized electron accumulation at the distorted tetrahedral structure.

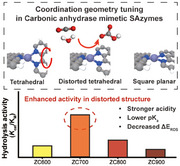

## 1 Introduction

Single‐atom nanozymes (SAzymes) have gained attention as potential biomimetic catalysts, emulating the structural characteristics of metalloenzymes with the desired catalytic activity,^[^ ^1^, ^2^, ^3^ ^]^ as well as bioorthogonal catalysts that do not occur in biological processes.^[^ ^4^, ^5^ ^]^ In particular, as cost‐effective alternatives to natural enzymes, SAzymes provide advantages in terms of production cost, designer capability, and high thermal and mechanical stability; thus, they are suitable for applications such as biosensing, therapeutics, environmental remediation, in vivo reactive oxygen species regulation, and biomedical fields.^[^ ^6^, ^7^, ^8^, ^9^, ^10^, ^11^ ^]^ Among various biomimetic SAzymes, those composed of nitrogen‐coordinated metal atoms on carbonaceous supports (M–NC, M: metal) have been extensively studied due to their high similarity to M–N_X_ sites in metalloenzymes.^[^ ^1^, ^12^, ^13^, ^14^ ^]^ These M–NC SAzymes have demonstrated unique enzyme‐like catalytic activities similar to those of horseradish peroxidase (HRP), cytochrome P450, and catalase.^[^ ^15^, ^16^, ^17^, ^18^ ^]^ However, creating SAzymes with precisely tuned molecular architectures remains challenging, and developing a reliable method to accurately reproduce the configurations of the enzyme active sites, which are constructed by myriad combinations of amino acid sequences, is essential.

Previous research has explored various strategies to modulate the activity of M–NC SAzymes, including modification of the atomic metal center and the local environment.^[^ ^15^, ^19^, ^20^ ^]^ For instance, Jiao et al. demonstrated that the type of metal center significantly affects the catalytic hydroperoxidation activity of planar M–NX SAzymes.^[^ ^21^ ^]^ Ji et al. enhanced the peroxidation kinetics through phosphorus substitution at the planar Fe–N_3_P site, highlighting the influence of coordinating ligand properties on catalysis.^[^ ^22^ ^]^ Additionally, Liu and colleagues introduced an axial N ligand to the planar Fe–N_4_ active site, which imitated the penta‐coordinated Fe center of HRP, and showed the effect of the surrounding environment on SAzyme activity.^[^ ^13^ ^]^ These M–N_4_ SAzymes, structured on carbonaceous scaffolds with a square‐planar coordination, mimicking the active center configuration of heme in heme enzymes. These studies confirm the essential role of the coordination environment in determining SAzyme activity. However, the effect of nonplanar active site configurations on catalytic activity remains underexplored.^[^ ^15^, ^23^ ^]^ This gap highlights the pressing need to develop SAzymes with controlled geometric configurations, with the aim of diversifying their catalytic functions.

**Figure 1:**
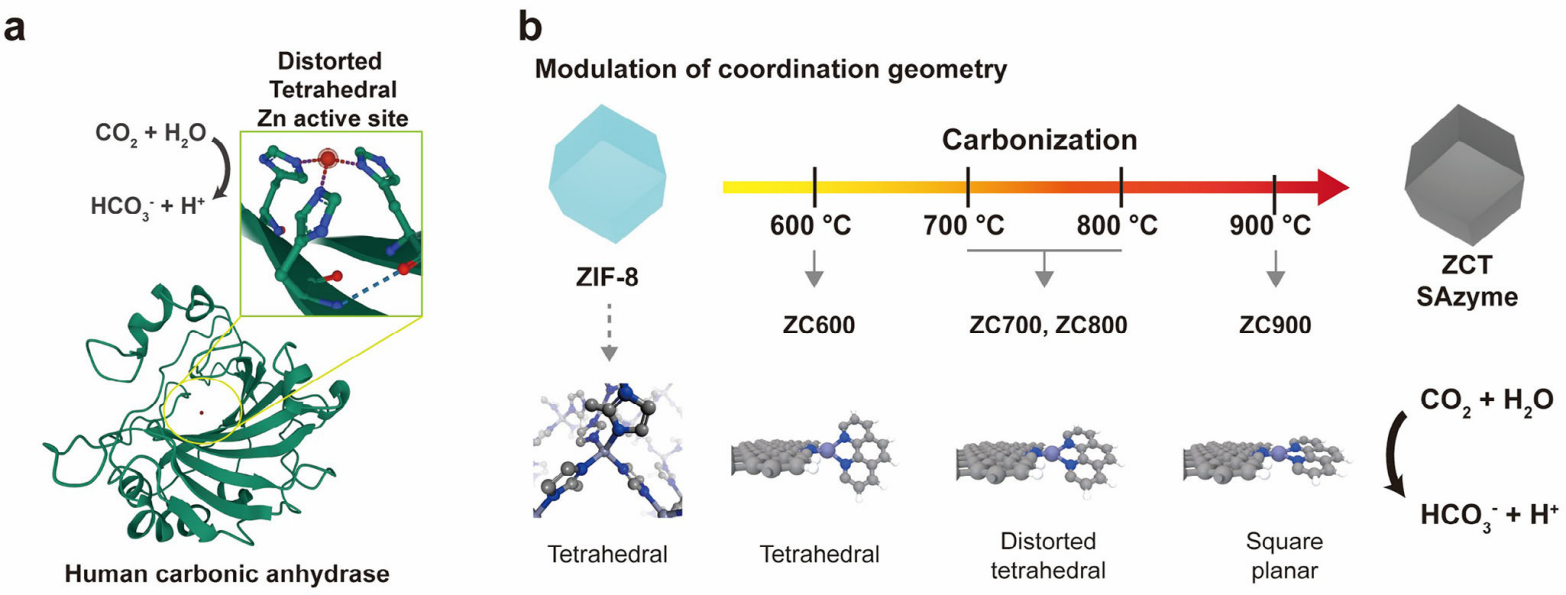
Scheme of the coordination geometries of Zn–N4 in ZCT SAzymes controlled by the carbonization of ZIF‐8. a) Structure of human CA showing a zinc active center that can convert carbon dioxide to bicarbonate and protons. b) Temperature‐controlled pyrolysis of ZIF‐8 to modulate the coordination geometry of Zn–N_4_ in ZCT SAzymes from tetrahedral to distorted tetrahedral and then to square planar structures to effectively mimic the nonplanar structure of the active site in CA.

Carbonic anhydrase (CA) is a well‐known metalloenzyme that plays a pivotal role in the reversible hydration of carbon dioxide into bicarbonate ion (HCO_3−_), facilitating CO_2_ transport in biological systems. It also demonstrates efficient esterase activity, making it a subject of significant interest in various applications, including biotechnology, large‐scale CO_2_ sequestration, biofuel production, and calcite synthesis.^[^ ^24^, ^25^, ^26^, ^27^, ^28^ ^]^ Extensive X‐ray crystallography studies have revealed that the active site of human CA features a Zn atom coordinated with three histidine imidazoles and a hydroxide in a distorted tetrahedral geometry, characterized by nonstandard Zn─His bond angles and varying bond lengths.^[^ ^29^ ^]^ This indicates that nature favors a distorted tetrahedral configuration, which could guide the enhancement of the catalytic activity of CA‐mimetic SAzymes by altering the coordination geometry. In this study, we synthesized CA‐mimetic SAzymes by carbonizing ZIF‐8 at various temperatures (ZCT), thereby controlling the coordination geometry of the Zn active site through pyrolysis (**Figure** **1**). The resulting ZCT SAzymes featured multiple Zn–N_4_ active sites on N‐doped carbon supports. As the pyrolysis temperature increased, the coordination geometry of the Zn center transformed from tetrahedral to distorted tetrahedral and ultimately to square‐planar. The ZCT SAzyme with the distorted tetrahedral configuration exhibited enhanced Lewis acidity and hydrolysis activity. Density functional theory (DFT calculations confirmed that the distorted coordination geometry leads to the localization of electron density, enhancing Lewis acidity and promoting the rate‐determining deprotonation step of Zn–H_2_O to Zn–OH, thus facilitating CO_2_ hydration.

## 2 Results and Discussion

### 2.1 Characterization of the ZCT SAzymes

**Figure 2:**
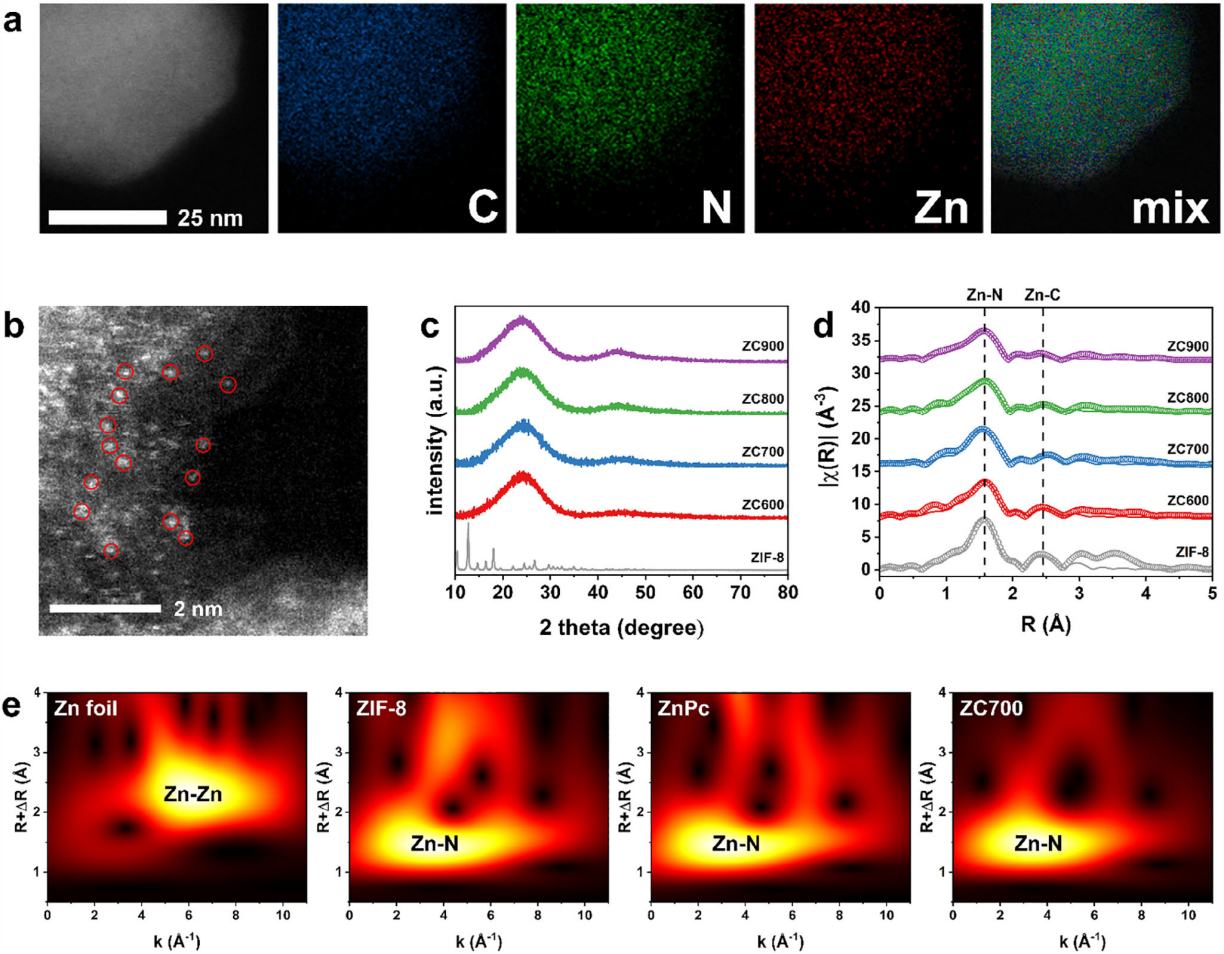
Characterization of the ZCT SAzymes. a) TEM and atomic mapping images and b) HAADF‐STEM image of ZC700. c) XRD patterns and d) FT‐EXAFS spectra and best fits in the R‐space of ZIF‐8 and ZCT SAzymes prepared at 600–900 °C. e) Wavelet‐transformed EXAFS contour plots of Zn foil, ZIF‐8, ZnPc, and ZC700.

ZIF‐8 is a well‐known precursor for producing square‐planar Zn–N_4_ single‐atom catalysts, which are formed through the gradual transformation of its original tetrahedral Zn–N_4_ configuration during pyrolysis at temperatures above 900 °C.^[^ ^30^, ^31^ ^]^ While the resulting Zn active sites in pyrolyzed ZIF‐8 retain an additional N ligand compared to natural CAs, they nevertheless serve as suitable models to explore the relationship between coordination geometry and catalytic performance,^[^ ^32^, ^33^, ^34^, ^35^, ^36^ ^]^ particularly for mimicking metalloenzymes with nonplanar active sites. The as‐prepared ZIF‐8 and ZCTs had a rhombic dodecahedral structure, as evident in the transmission electron microscopy (TEM) images (Figure S1, Supporting Information). The size (d = ∼60 nm) and polyhedral structure of ZIF‐8 were preserved even after pyrolysis at 900 °C. TEM‐energy‐dispersive X‐ray spectroscopy (EDS) analysis confirmed the even distribution of Zn atoms on the carbonaceous nanoparticles without crystalline Zn clusters, regardless of the pyrolysis temperature (**Figure** **2**a; Figure S2, Supporting Information). Aberration‐corrected high‐angle annular dark‐field scanning TEM (HAADF‐STEM) further revealed single Zn atoms in the ZC700 SAzyme, as indicated by the presence of isolated bright dots (marked by red circles) on the support (Figure 2b). The X‐ray diffraction (XRD) patterns of the ZCT SAzymes displayed two broad peaks corresponding to the (002) and (100) carbon planes at 2θ = 26° and 44°, respectively (Figure 2c). These XRD patterns showed a contrast between the weakly crystalline nature of the N‐doped carbon and the highly crystalline ZIF‐8, indicating that the organized Zn‐mIm coordination collapsed during thermal treatment. In addition, no sharp peaks associated with crystalline Zn^0^ particles or ZnO were observed; this result was consistent with the above TEM observations. Notably, acid washing successfully removed the unstable crystalline Zn species detected from the non‐acid‐treated ZCTs (Figures S3 and S4, Supporting Information), while the Zn species remained in a single‐atom state.^[^ ^37^, ^38^ ^]^ The pyrolysis process of ZIF‐8 was examined using thermogravimetric analysis (TGA) (Figure S5, Supporting Information). ZIF‐8 exhibited good thermal stability up to 500 °C, with significant mass decreases at 608 and 640 °C. These decreases were attributed to the release of free methyl groups and CN^−^/HCN groups from imidazole rings, respectively.^[^ ^37^ ^]^ The organic linker molecule of mIm prevented the aggregation of the Zn ions, assisting the formation of single‐atom Zn sites on N‐doped carbon supports. The bond between Zn^2+^ and imidazole is the most stable among N‐donor ligands, which may minimize Zn release during the pyrolysis process.^[^ ^39^, ^40^ ^]^ The Zn loadings determined by inductively coupled plasma‐optical emission spectroscopy (ICP‒OES) were 4.96, 5.41, 6.96, and 5.99 wt.% for ZC600, ZC700, ZC800, and ZC900, respectively. The Zn content increased beyond 600 °C due to the enhanced loss of organic mass but decreased at 900 °C as Zn evaporated at ≈906 °C. The N_2_ isotherms of ZIF‐8 and the ZCTs were analyzed to determine their porosity and Brunauer‒Emmett‒Teller (BET) surface area (Figure S6 and Table S1, Supporting Information). ZIF‐8 exhibited a typical reversible type‐I isotherm, which transitioned to type‐II with a hysteresis loop attributed to capillary condensation after carbonization.^[^ ^41^, ^42^ ^]^ This result indicated significant changes in the porous texture during the pyrolysis process. All samples exhibited high gas adsorption at p/p_0_ < 0.1, indicating a microporous structure. The surface area and pore volume significantly decreased after the carbonization of ZIF‐8. After thermal treatment, the microporous structure of ZIF‐8, with a pore width of ∼1.1 nm, collapsed, and new micropores with pore sizes of 0.5 nm and 1–2 nm were generated.

The chemical composition of the ZCT was examined by X‐ray photoelectron spectroscopy (XPS) (Figure S7 and Table S2, Supporting Information).^[^ ^43^, ^44^ ^]^ High‐resolution C1s spectra showed that the carbon textures of ZCT included four distinct C species located at 284.6, 285.8 (± 0.1), 287.5 (± 0.1) and 288.8 (± 0.2) eV, attributed to C─C/C═C, C═N, C─N, and COOH bonds, respectively. The proportion of C═N bonds decreased as the carbonization temperature increased due to the evaporation of nitrogen. The deconvolution of the N 1s spectra revealed the presence of four N species in ZCT: pyridinic N (398.3 ± 0.1 eV), pyrrolic N (400.0 ±0.1 eV), graphitic N (400.9 ± 0.1 eV), pyridine‐N‐oxide (402.5 ± 0.1 eV), and chemisorbed N‐oxide (404.2 ± 0.1 eV). For ZC600, pyridinic N and pyrrolic N were the dominant species, and the proportions of graphitic N and N‐oxide species gradually increased with increasing pyrolysis temperature. High‐resolution Zn 2p spectra of the ZCT samples revealed that the oxidation state of Zn was maintained at a divalent state (Figure S7g,h, Supporting Information). The slight peak shift of Zn 2p_3/2_ from 1021.1 to 1021.4 eV with increasing carbonization temperature indicated a change in the coordination environment of the Zn species in ZCT.^[^ ^45^ ^]^ The carbonaceous support of the ZCT SAzyme was further analyzed by Raman spectroscopy (Figure S8, Supporting Information). The spectra of ZCT SAzymes exhibited two broad D (∼1335 cm^−1^) and G (∼1587 cm^−1^) bands and featureless second‐order bands (2D and G + D) between 2700 and 3000 cm^−1^.^[^ ^46^ ^]^ The D band is known as the disorder band or defect band, while the G band, known as graphitic band, is attributed to planar configuration of sp^2^ carbon. The I_D_/I_G_ ratios were 0.92, 1.05, 1.10, and 1.14 for ZC600, ZC700, ZC800, and ZC900, respectively. This result indicates that the defects increase with the increase of the carbonization temperature, presumably because of the evaporation of N species resulting in defects on carbonaceous support.^[^ ^47^ ^]^ At the same time, the portion of pyrrolic N decreases and graphitic N increases with the carbonization temperature (Figure S7j, Supporting Information). The lengths of C─N bonds of pyrrolic N and graphitic N are 1.33–1.34 Å and 1.41 Å, respectively, while the in‐plane C─C bond length is 1.42 Å.^[^ ^48^, ^49^ ^]^ The bigger difference of the bond length between C─N bond and C─C bond of pyrrolic N than that of graphitic N might induce compressive strain to the carbonaceous matrix. Therefore, the geometry of the Zn‐N_4_ active sites of ZCT SAzymes might undergo flattening as the carbonization temperature increases, distorting the original tetrahedral configuration of ZIF‐8. Meanwhile, in the ZC600 SAzyme, additional bands of ─CH_3_ bending/C–N stretching and ring stretching vibrations were observed at 1425 and 1510 cm^−1^, respectively. These peaks indicated that the methyl groups and ring structure of the N‐containing carbon oligomers remained even after pyrolysis at these temperatures.^[^ ^50^, ^51^ ^]^ The remaining organic components were further confirmed by FT‐IR analysis (Figure S9, Supporting Information). The spectrum of ZIF‐8 was consistent with that in previously reported literature.^[^ ^52^, ^53^ ^]^ After the pyrolysis of ZIF‐8, the C‐H bending peak at 1385 cm^−1^ from the methyl group was observed in ZC600, and this peak disappeared above 600 °C. Furthermore, the bands of the ═C─H and C─N bonds of ZIF‐8 at 1146 and 1177 cm^−1^ shifted to 1120 and 1144 cm^−1^, respectively, in ZC600 and ZC700 and disappeared in ZC800. The band corresponding to the C─H bond at 619 cm^−1^ also disappeared at 800 °C, indicating almost complete carbonization of the mIm ligands.

X‐ray absorption spectroscopy (XAS) analyses were further conducted to elucidate the state of the single‐atom Zn on the carbon support. The Fourier‐transformed extended X‐ray absorption fine structure (FT‐EXAFS) spectra of the ZCT samples and their best fits revealed the coordination structure of the Zn species (Figure 2d). Two peaks in the FT‐EXAFS spectra at 1.54 and 2.57 Å were assigned to the Zn─N and Zn─C paths, respectively, while the Zn–Zn path was not observable. The fitting results from **Table** **1** indicated that Zn existed in the Zn–N_4_ coordination structure, showing that the Zn–N_4_ coordination remained after carbonization. Remarkably, the Zn─N bond length gradually decreased from 2.046 to 2.029 Å as the carbonization temperature increased from 600 to 900 °C. Thus, the coordination structure could be precisely controlled at the atomic level by adjusting the pyrolysis temperature. Wavelet‐transformed EXAFS contour plots of ZC700 and Zn references (Zn foil, ZIF‐8, and Zn‐phthalocyanine (ZnPc)) further confirmed the absence of a Zn–Zn path centered at k = ∼6.1 Å^−1^ and R + ΔR = ∼2.3 Å (Figure 2e; Figure S10, Supporting Information).

**Table 1:** Zn‐EXAFS fitting results of the ZIF‐8 and ZCTs.

	Path	N^a)^	Distance [Å]	Debye‐Waller factor [Å^2^]	R‐factor^b)^
**ZIF‐8**	Zn‐N	4.24(0.32)	1.994(0.006)	0.004(0.001)	0.008
	Zn‐C	4.11(0.56)	2.984(0.017)	0.004(0.001)	
**ZC600**	Zn–N	4.31(0.39)	2.046(0.009)	0.007(0.002)	0.010
	Zn–C	1.83(0.47)	3.009(0.028)	0.002(0.001)	
**ZC700**	Zn–N	4.26(0.54)	2.038(0.012)	0.007(0.002)	0.015
	Zn–C	3.41(0.43)	3.010(0.037)	0.007(0.002)	
**ZC800**	Zn–N	4.46(0.26)	2.034(0.005)	0.009(0.001)	0.004
	Zn–C	1.18(0.25)	2.977(0.021)	0.002(0.001)	
**ZC900**	Zn–N	4.30(0.26)	2.029(0.006)	0.010(0.001)	0.004
	Zn–C	1.01(0.25)	2.911(0.028)	0.004(0.001)	

^a)^ Coordination number;

^b)^ a measure of the mean square sum of the misfit at each data point. Fit range: 2.2 < k < 12 Å^−1^; 1 < R < 2.8 Å; Fit window: Hanning. The values in brackets indicate the error ranges.

**Figure 3:**
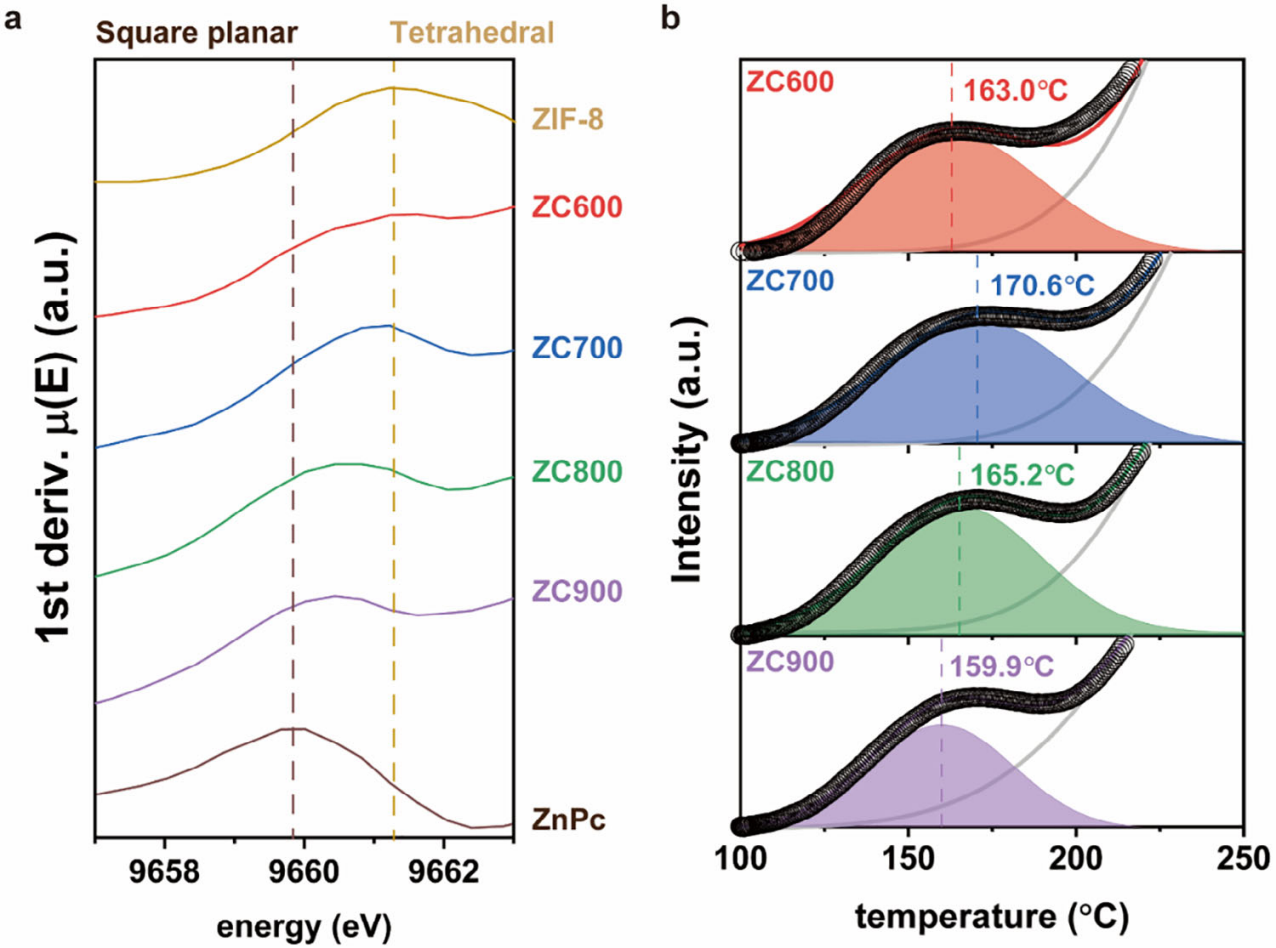
Variations in the coordination geometry and acidity with increasing carbonization temperature. a) 1st derivatives of the Zn‐XANES spectra of ZC600, ZC700, ZC800, ZC900 and Zn references (ZIF‐8 and ZnPc) and b) NH_3_‐TPD profiles of ZCT.

Zn^2+^ has an electronic configuration of [Ar]3d^10^. The filled d shell does not provide crystal field stabilization of Zn^2+^. Therefore, the coordination geometry of Zn is determined by the ligand environment.^[^ ^54^, ^55^ ^]^ In a given ligand system, the electronic structure of Zn–N_4_ is determined by the coordination geometry. The X‐ray absorption near‐edge structure (XANES) spectrum is used to identify the detailed coordination geometry of Zn–N_4_ in the ZCT SAzymes because the XANES region provides K‐absorption edge energy and 1s→4p electronic transition (**Figure** **3**a; Figure S11, Supporting Information).^[^ ^56^, ^57^, ^58^ ^]^ The K‐absorption edge energy of Zn was determined from the first maximum of the 1st derivative of the XANES spectrum. A Zn reference of ZnPc, with a square‐planar coordination geometry, has the highest K‐absorption edge energy at 9659.9 eV, while another reference of ZIF‐8, with a well‐defined tetrahedral geometry, has a maximum at 9661.2 eV. Remarkably, the K‐absorption edge energy of the ZCT SAzymes changed with respect to the carbonization temperature. Since ZnPc, ZIF‐8, and ZCT have the same oxidation states of +2 and Zn–N_4_ coordination, the difference in the XANES spectra was caused by the variation in the coordination geometry. As shown in Figure 3a, the first maximum of the 1st derivative of the XANES spectra of the ZCT SAzymes gradually shifted from the position of ZIF‐8 to that of ZnPc with increasing temperature, showing the transformation of the coordination geometry. ZC600 had a similar K‐absorption edge energy to that of ZIF‐8, indicating that its coordination geometry did not significantly deviate from the tetrahedral configuration of ZIF‐8. The K‐absorption edge energy of ZC900 was 9660.4 eV, which was close to that of ZnPc with a square‐planar configuration. The K‐absorption edge energy maxima of ZC700 and ZC800 were between those of the tetrahedral and square‐planar configurations, indicating distorted tetrahedral Zn–N_4_ configurations. By considering both the EXAFS and XANES results, the distance and geometry of the Zn coordination with ligating N species were changed by modulating the pyrolysis temperature. This could subsequently alter the electronic density distribution at the Zn center and neighboring ligands, affecting the reactivity.^[^ ^59^, ^60^ ^]^

Since the distortion in the coordination geometry could exhibit strong Lewis acidity due to the facilitated formation of vacant positions in the coordination sphere,^[^ ^61^ ^]^ the Lewis acidity of ZCT SAzymes was examined as further evidence of the transformation in the coordination geometry. NH_3_‐temperature programmed desorption (NH_3_‐TPD) was conducted to investigate the acidity of the ZCT SAzymes (Figure 3b; Figure S12, Supporting Information). Two desorption peaks were identified from the deconvolution of the NH_3_‐TPD profile. The peak at lower temperatures was attributed to the presence of Lewis acidic sites, i.e., Zn^2+^, while the other peak was caused by the presence of the Brønsted acid sites, such as protonated pyrrolic N groups in the N‐doped carbon scaffold. The peak maxima of the Lewis acidic site were located at 163.0, 170.6, 165.2, and 159.9 °C for ZC600, ZC700, ZC800, and ZC900, respectively. Based on these NH_3_ desorption temperatures, the acidities of the Zn centers were in the order of ZC700 > ZC800 > ZC600 (tetrahedral) > ZC900 (square‐planar). This order of Lewis acidity indicated that ZC700 had the most distorted structure among the tested ZCT SAzymes, while ZC600 and ZC900 had more uncurled structures. Furthermore, since the Lewis acidity of the zinc ions is an important factor that promotes CO_2_ hydration by lowering the pK_a_ of the Zn‐bound water,^[^ ^62^, ^63^ ^]^ the ZC700 and ZC800 SAzymes with distorted tetrahedral geometries are expected to be more advantageous for CO_2_ hydration.

### 2.2 Ab Initio Computations of the Catalytic Activity of ZCT

**Figure 4:**
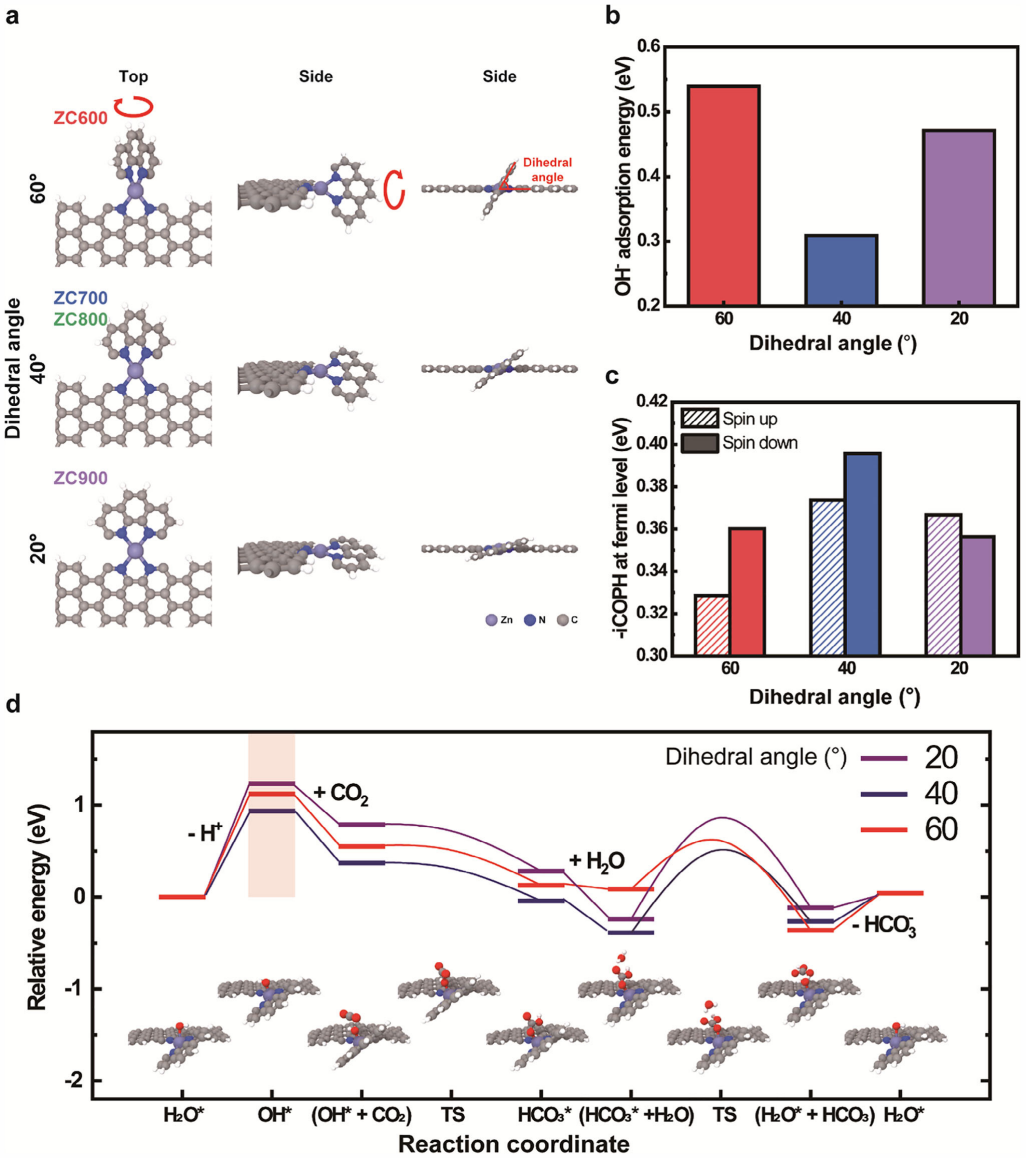
Computed catalytic activity of the ZCT SAzyme. a) Model systems of ZC600, ZC700/ZC800 and ZC900. The model systems exhibit dihedral angles of 60°, 40°, and 20° for ZC600, ZC700/ZC800, and ZC900, respectively. b) Computed OH^−^ adsorption energies at pH 14 and c) Integrated crystal orbital Hamilton population (‐iCOHP) under various the dihedral angles. d) Energy diagram of CO_2_ hydration as a function of the dihedral angle at pH 14. The gray, blue, red, and white circles represent C, N, O, and H, respectively.

Ab initio computations revealed that the catalytic activity was strongly influenced by the geometry of the Zn–N_4_ active center, as illustrated in **Figure** **4**. To investigate the effect of the coordination geometry on the CO_2_ hydration activity, we constructed model systems by combining Zn–N_4_, hydrogen‐passivated graphene, and 1,10‐phenanthroline (Figure 4a). The model systems exhibited specific dihedral angles of 20°, 40°, and 60° when rotating 1,10‐phenanthroline. These angles were selected to represent the square planar (ZC900), distorted tetrahedral (ZC700 and ZC800), and tetrahedral (ZC600) geometries, respectively. The model systems served to explicitly study the effect of the coordination geometry (dihedral angles) on the catalytic activity. The bond lengths of Zn─N were identical among all the model systems, and all other atoms were allowed to fully relax.

The rate‐determining step in CO_2_ hydration catalyzed by CA is the deprotonation of Zn–H_2_O to form Zn–OH,^[^ ^25^, ^64^ ^]^ which depends on the acidity of the active center. Therefore, we calculated the OH^−^ adsorption energy for each Zn–N_4_ geometry, which served as a measure of the acidity of the active center (Figure 4b; Figure S13, Supporting Information). The distorted tetrahedral geometry with a dihedral angle of 40° showed the lowest adsorption energy among the three geometries. The reduction of the adsorption energy in the distorted structure remained consistent even when a model system with a shorter Zn─N bond length was considered, aligning with the experimental results (Figure S14, Supporting Information). The tendency to have the lowest adsorption energy at an angle of 40° remained even at an elevated pH of 14, although the adsorption energy steadily decreased with pH (Figure S15, Supporting Information). Theaverage integrated crystal orbital Hamilton populations (‐iCOHP) obtained by integration of the COHP (Figure S16a, Supporting Information) represent the chemical bond strength between two atoms at the Fermi level by projecting precalculated plane wave functions to Slater‐type orbitals. The ‐iCOHP for the spin‐up and spin‐down population pairs at the Fermi level were determined to be (0.356 eV, 0.367 eV), (0.374 eV, 0.396 eV), and (0.360 eV, 0.329 eV) for the Zn–N_4_ geometries at the dihedral angles of 60°, 40°, and 20°, respectively (Figure 4c). The highest ‐iCOHP at the dihedral angle of 40° confirmed the pronounced Zn–O affinity in the distorted tetrahedral geometry. In addition, the average bond strengths between Zn and each coordinating N atom in the Zn–N_4_ configurations, depending on the geometry, were consistently computed at 0.55 eV, as depicted in Figure S16b (Supporting Information). In Zn–N_4_ structures with dihedral angles of 20° and 40°, electrons locally accumulated in the region between Zn and O, as illustrated in Figure S17 (Supporting Information). However, at 60°, the electrons were more widely distributed due to steric hindrance, inhibiting the stabilization of OH^−^ on the Zn center. The localized accumulation of electrons between Zn and O signified a strong bond, rationalizing the high OH^−^ adsorption energy.

The calculated energy diagram of the CO_2_ conversion reaction also exhibited differences according to the coordination geometry of the Zn active center (Figure 4d). The H^+^ ion dissociates from H_2_O at the Zn active center, forming OH^−^. The free CO_2_ molecule then reacts with the OH^−^ on the Zn center, leading to the formation of HCO_3_* with minimal activation barriers of 0.02, 0.01, and 0.04 eV for the 20°, 40°, and 60° model systems, respectively (Figure S18a, Supporting Information). The substitution of HCO_3_* with H_2_O further proceeds on the Zn center with activation barriers of 1.1, 0.90, and 0.53 eV for the 20°, 40°, and 60° geometries, respectively, subsequently generating HCO_3−_ (Figure S18b, Supporting Information). Under the charge conservation condition applied in our simulations, the adsorbed bicarbonate species (HCO_3_^*^) on Zn is assumed to possess a negative charge, which indicates HCO_3_^*^ on Zn and the free HCO_3_^−^ have the same net charge. As expected, the rate‐determining step involved the deprotonation of adsorbed H2O on Zn, with the computed reaction energies of 1.12, 0.94, and 1.23 eV corresponding to Zn–N_4_ geometries at dihedral angles of 60°, 40°, and 20°, respectively (shadowed area in Figure 4d). This trend in the reaction energy of the rate‐determining step aligns well with the computed OH^−^ adsorption energy in Figure 4b. The deprotonation reaction energy decreased with increasing pH, which aligned with the facilitated CO_2_ hydration under basic conditions (Figure S19, Supporting Information). The deviation in the order of H_2_O adsorption in Figure S20 (Supporting Information) from the deprotonation energy trend in Figure 4d explains the stability of OH^−^ on Zn, highlighting the role of active site acidity in governing the dehydrogenation reaction. These computational results suggest that modifying the acidity of the active center through coordination geometry tuning can enhance CO_2_ hydration activity by promoting the deprotonation of Zn‐bound H_2_O.

### 2.3 Catalytic Hydrolysis Activity of ZCT

**Figure 5:**
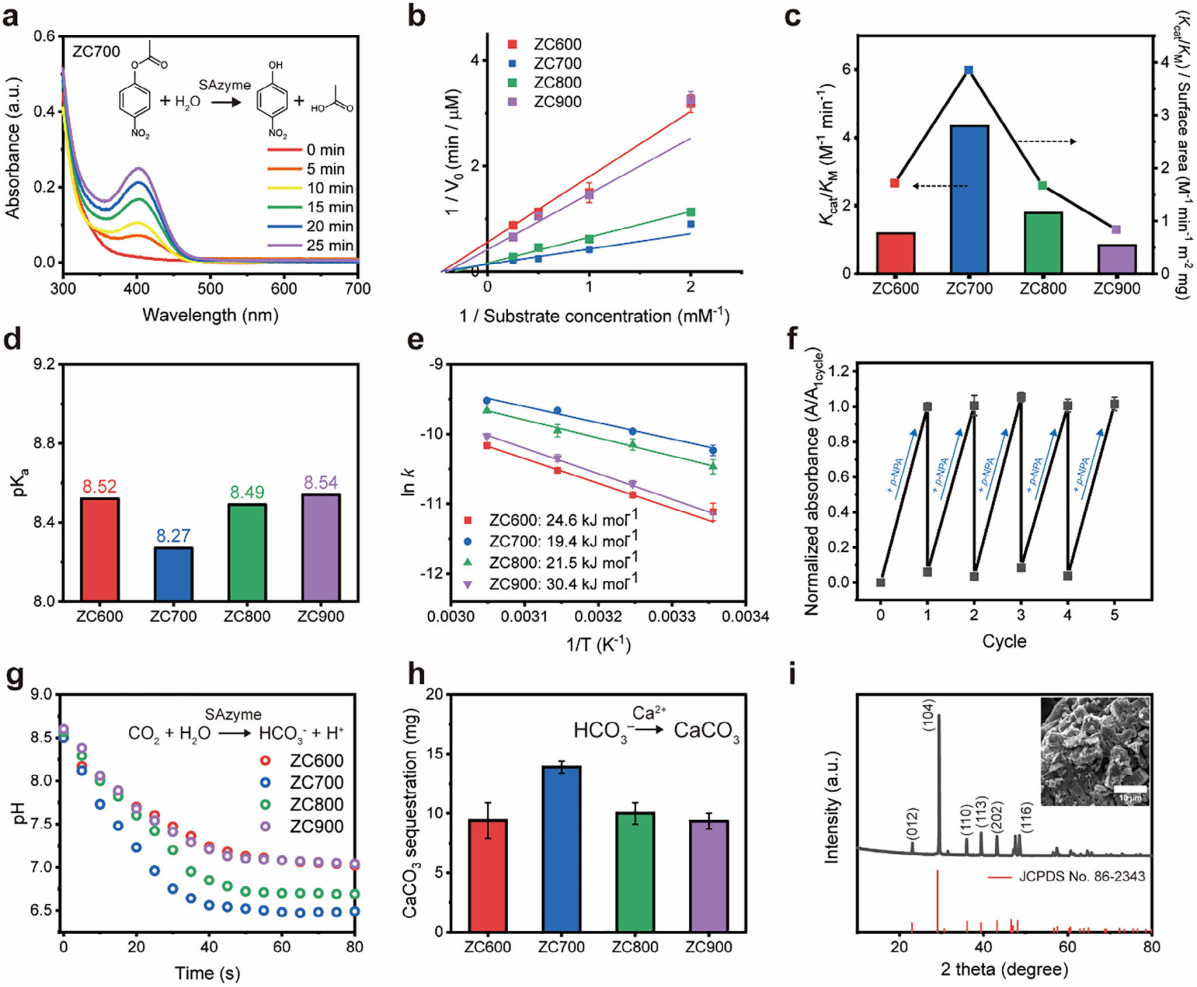
Catalytic hydrolysis activity and CO_2_ sequestration of the ZCT SAzyme. a) UV‒Vis absorbance spectra of *p*‐nitrophenol (*p*‐NP) produced by the catalytic hydrolysis of *p*‐nitrophenyl acetate (*p*‐NPA) using ZC700 SAzyme (1 mg mL^−1^). The gradual increase in the peak of *p*‐NP (at 400 nm) indicates the catalytic activity of ZC700 SAzyme. b) The double reciprocal plot of the substrate‐dependent kinetics of the ZCT SAzymes. c) Catalytic efficiencies (*K*_cat_/K_m_) and catalytic efficiency/surface area (*K*_cat_/K_m_ ∙ surface area) of ZCT SAzymes. d) pK_a_ values of the ZCT SAzymes measured from the pH profiles of the *p*‐NPA hydrolysis activity. e) Arrhenius plots and activation energies (E_a_) of the ZCT SAzymes. f) Reusability test of the ZC700 catalyst for *p*‐NPA hydrolysis. The normalized absorbance represents the measured absorbance of *p*‐NP (at 400 nm) normalized to the absorbance of *p*‐NP acquired from the 1st cycle of the catalytic reaction. g) Profiles of the pH decay representing the catalytic CO_2_ hydration activity of the ZCT SAzymes. h) Weights of the CaCO_3_ precipitates produced after CO_2_ hydration using the ZCT SAzymes. i) XRD profile of the CaCO_3_ precipitates (inset: SEM images of CaCO_3_ precipitates).

Similar to the active site of CA, the Zn center of ZCT exhibited hydrolytic activity, as demonstrated by the hydrolysis of *p*‐NPA to *p*‐NP (**Figure** **5**a; Figure S21, Supporting Information).^[^ ^65^, ^66^ ^]^ All ZCT SAzymes hydrolyzed *p*‐NPA, with ZC700 exhibiting the highest hydrolysis activity, showed marked increases in the *p*‐NP production over time (Figure S22, Supporting Information). The activities of ZCT SAzymes followed the order of ZC700 > ZC800 > ZC600 ≈ ZC900.

The enzyme‐like activity of ZCT is well illustrated in the kinetic study of the *p*‐NPA hydrolysis.^[^ ^67^, ^68^ ^]^ The Michaelis–Menten kinetics model was applied to describe the catalytic reaction. As illustrated in Figure 5b and Figure S23 (Supporting Information), the Michaelis‐Menten model fits well to describe the reaction kinetics, verifying the enzyme‐like behavior of ZCT. The catalytic efficiencies (*K*_cat_/K_m_) of ZCT SAzymes were calculated from their turnover number (*K*_cat_) and Michaelis constant (K_M_), following the order of ZC700 > ZC800 > ZC600 > ZC900. The surface area‐normalized catalytic efficiencies, excluding the surface area effect, also followed a similar order (Figure 5c and Table S3, Supporting Information). The hydrolysis activity of ZCT SAzymes correlates with the trends in Lewis acidity determined earlier. In addition, the surface‐normalized catalytic efficiency of ZC700 was 2.38 times higher than that of ZIF‐8, while its catalytic efficiency was 5.43 times higher than that of ZnPc in our experimental condition, with ZIF‐8 and ZnPc exhibiting Zn–N_4_ geometries with dihedral angles of 90° and 0°, respectively (Figure S24 and Table S3, Supporting Information). These results were in agreement with the Lewis acidity of ZCT SAzymes investigated earlier by the NH_3_‐TPD experiment and DFT calculations (Figures 3b and 4b). Given that *p*‐NPA hydrolysis is an acid‐catalyzed reaction,^[^ ^63^ ^]^ the high Lewis acidity of ZC700, attributed to its distorted tetrahedral geometry, was responsible for its enhanced hydrolysis, suggesting that tuning coordination geometry is advantageous for improving catalytic activity, while the N species or the I_D_/I_G_ ratio seems to have less impact (Figure S25, Supporting Information). The higher catalytic activity of ZC700 was supported by other physicochemical properties. First, the pK_a_ values of the water bound to Zn active sites in ZCT SAzymes were experimentally evaluated. The rate of *p*‐NPA hydrolysis dramatically increased with increasing pH (Figure S26, Supporting Information) and was attributed to the increase in pK_a_.^[^ ^66^, ^69^, ^70^ ^]^ The calculated pK_a_ of ZC700 (8.27) was lower than those of other ZCT SAzymes; thus, ZC700 was advantageous for the deprotonation of Zn–bound H_2_O to form nucleophilic Zn–OH (Figure 5d). The activation energy (E_a_) of the ZCT SAzyme is another property indicative of its catalytic activity. Assuming a 1st‐order reaction, the reaction rate constant (k) was determined from the initial reaction rate at controlled temperatures (Figure 5e). ZC700 had the lowest activation energy of 19.4 kJ mol^−1^, while other ZCT SAzymes had higher values in the order of ZC800 (21.5 kJ mol^−1^) < ZC600 (24.6 kJ mol^−1^) < ZC900 (30.4 kJ mol^−1^). This result indicates that the geometric configuration plays a crucial role in determining the thermodynamic reaction barrier. The catalytic performance of ZC700 is compared with native enzymes and their mimics in Table S4 (Supporting Information). Although ZC700 showed lower catalytic activity than the native CAs, the above results demonstrate that the catalytic activity of the enzyme‐mimetic catalysts can be improved by the geometric tuning of the active site, which has been overlooked by previous research on enzyme‐mimetics.

The reusability of ZC700 SAzyme maintained 99% of its initial activity even after five cycles of reuse (Figure 5f). Due to its maintenance of catalytic activity, the deterioration of the ZCT, such as the leakage of Zn from the N‐doped carbon support, was considered to be negligible. XANES and EXAFS analyses of the used ZC700 were conducted to confirm that the original geometric structure of the Zn‐N_4_ active site was maintained after the catalytic reactions (Figure S27, Supporting Information). The used ZC700 exhibited slightly reduced 1s→4p electronic transition and slightly increased white line intensity, while maintaining the white line position. This indicates the coordination number increased, accompanying the slight position shift of the K‐edge energy to higher energy.^[^ ^71^ ^]^ FT‐EXAFS analysis further revealed that the coordination number of Zn─N/O bond increased to 5.49 after the reaction (Table S5, Supporting Information). The increase in the coordination number is presumably attributed to the addition of ─OH group to Zn–N_4_ active site after the catalytic reaction. Nevertheless, the results of the reuse test suggest that the original distorted tetrahedral geometry of Zn–N_4_ is maintained under the reaction conditions.

To demonstrate practical application, catalytic CO_2_ sequestration by the ZCT SAzyme was conducted. First, the pH reduction of the CO_2_‐saturated solution was monitored in the presence of ZCT SAzymes over time (Figure 5g). In accordance with *p*‐NPA hydrolysis, ZC700 exhibited the fastest pH decay among all ZCT SAzymes and reached the lowest pH after 80 s of reaction. CO_2_ sequestration was subsequently conducted via a reaction with Ca^2+^ ions to mineralize CaCO_3_. In agreement with the CO_2_ hydration results, the largest amount of CaCO_3_ calcite was precipitated from the ZC700‐treated CO_2_ solution (Figure 5h). XRD and elemental analysis verified the high purity of the calcite structure (Figure 5i; Figure S28, Supporting Information).

## 3 Conclusion

In summary, based on the experimental data and DFT calculations of the CA‐mimetic Zn–N_4_ SAzymes, variations in their coordination geometries altered the electron density distribution, Lewis acidity, and subsequent hydrolysis activity of the Zn active center. These changes could be simply achieved by controlling the pyrolysis temperature during the SAzyme preparation. Among the Zn–N_4_ active sites with different coordination geometries, the distorted tetrahedral geometry exhibited the highest catalytic activity. Its asymmetric coordination structure caused a localized electron density distribution, leading to enhanced Lewis acidity of the Zn center. The temperature‐controlled pyrolysis of ZIF‐8 showed a way to modulate the coordination geometry, along with the production of N‐doped carbon support; this was difficult to achieve with the SAzymes implanted in pretreated carbon supports. Furthermore, the maintenance of enhanced catalytic activity even after repeated reactions indicated the outstanding stability of the single‐atom catalyst. The reaction kinetics followed an enzymatic process; and due to the Lewis acidity of the active center and the underlying reaction mechanism, these SAzymes are promising CA mimic analogs with the potential to be extended as analogs of hydrolases, such as organophosphate hydrolase.^[^ ^72^ ^]^ As one of the strategies to engineer the coordination environment,^[^ ^73^, ^74^ ^]^ this approach of controlling the geometric configuration of metal active centers can provide the basis for a de novo methodological approach to develop a more effective and extended series of natural enzyme mimics in the SAzyme field for future industrial and biological applications.

## Conflict of Interest

The authors declare no conflict of interest.

## Supporting information



Supporting Information

## Data Availability

The data that support the findings of this study are available in the supplementary material of this article.
