# Supplementary material for: Coordination Geometry Tuning in a Single‐Atom Nanozyme to Mimic Metalloenzymes with Nonplanar Active Site
Source: Adv Sci (Weinh). 2025 Aug 9;12(34):e05733. doi: 10.1002/advs.202505733 (PMC12442611; doi:10.1002/advs.202505733)
Supplement: Supplementary file 1 — Supporting Information [file ADVS-12-e05733-s001.doc]

Supporting Information

Coordination Geometry Tuning in a Single-atom Nanozyme to Mimic Metalloenzymes with Nonplanar Active Sites

Hyesung Lee,^[a],†^ Changjoon Keum,^[b],†^ Choah Kwon,^[c],†^ Sangtae Kim,^[c]^ Youngdo Jeong,*^[b,d]^ and Sang-Yup Lee*^[a]^

[a] Dr. H. Lee, Prof. S.-Y Lee
Department of Chemical and Biomolecular Engineering
Yonsei university
Seoul 03722, Republic of Korea
E-mail: leesy@yonsei.ac.kr
[b] Dr. C. Keum, Dr. Y. Jeong
Center for Advanced Biomolecular Recognition, Biomedical Research Division
Korea Institute of Science and Technology (KIST)
Seoul 02792, Republic of Korea
E-mail: zerodegree@kist.re.kr
[c] Dr. C. Kwon, Prof. S. Kim
Department of Nuclear Engineering and Department of Materials Science and Engineering
Hanyang University
Seoul 04763, Republic of Korea
[d] Dr. Y. Jeong
Department of HY-KIST Bio-convergence
Hanyang University
Seoul 04763, Republic of Korea
^†^These authors contributed equally.

**Experimental and computational details**

**Materials**

Zn(NO­_3_)_2_·6H_2_O (98%) and 2-methylimidazole (mIm, 99%) were purchased from Sigma-Aldrich. Methanol (99.8%) and 0.5 M H_2_SO_4_ were purchased from Duksan Chemical Co. All chemicals were used as received without additional purification.

**Synthesis of ZIF-8 and carbonized ZIF-8 (ZCT)**

For preparing the CA-mimetic SAzyme, ZIF-8 was used as a solid precursor. ZIF-8 was synthesized by mixing Zn(NO_3_)_2_·6H_2_O and mIm solutions in methanol at room temperature. Briefly, a volume of 150 mL of Zn(NO_3_)_2_·6H_2_O (3.949 g, 13.3 mmol) solution in methanol and the same volume (150 mL) of 2-methylimidazole (8.718 g, 106.2 mmol) solution in methanol were mixed for 1 h at room temperature. With the progress of the Zn-mIm coordination, white crystalline ZIF-8 particles were precipitated. The precipitates were collected by centrifugation (8,000 rpm, 20 min), washed with methanol thrice, and dried at 60 °C in a drying oven. The dried ZIF-8 was carbonized at various pyrolysis temperatures in the range of 600-900 °C with a ramping rate of 2 °C/min for 2 h under N_2_ environment. The carbonized ZIF-8 is labeled as ZCT where T represents the carbonization temperature. After the carbonization, the resulting black powder was soaked in 0.5 M H_2_SO_4_ at 80 °C for 8 h to remove metallic Zn species. The powder was collected by centrifugation (13,000 rpm, 30 min), washed with water thrice, and dried at 60 °C.

**Characterization**

The morphology of the ZCT was observed using a transmission electron microscope (TEM, JEOL-F200, JEOL). ZCT particles were dispersed in deionized water and dropped on the carbon-coated copper grid for the preparation of TEM samples. Aberration-corrected high-angle annular dark field-scanning transmission electron microscopic (HAADF-STEM) images were obtained using HR-TEM (JEM-ARM200F, JEOL, 200 kV). The crystalline structure of ZIF-8 and ZCT was analyzed from the X-ray diffraction patterns in the range of 5-80°. An X-ray diffractometer (XRD, Ultima IV, Rigaku) with a Cu Kα radiation source (λ = 1.54059 Å) is used for the XRD analysis. Raman spectra were recorded using a Raman spectrometer (LabRam Aramis, Horriba Jovin Yvon) equipped with a 532 nm ND:Yag laser. The porosity of ZCT was determined from the N_2_ adsorption-desorption isotherms using an automatic gas adsorption analyzer (BELSORP-max, MicrotracBEL Corp.). Surface area and pore volume were calculated by the multipoint Brunauer-Emmett-Teller (BET) analysis and t-method, respectively. The pore size distributions of ZCT were calculated using SAIEUS software with carbon-N2, NLDFT, and standard slit model.^50^ The Zn loading in ZCT SAzyme was determined using an inductively coupled plasma-optical emission spectrometer (ICP-OES, Optima 8300, Perkin Elmer). Analysis on the surface composition of the ZCT was conducted using an X-ray photoelectron spectrometer (K-alpha, Thermo Scientific Inc.) equipped with an Al Kα monochromated X-ray source (1486.6 eV) under the conditions of 50 scan numbers, 40 eV pass energy, and 0.5 eV scanning resolution. The obtained X-ray photoelectron spectra were corrected using the C1s peak at 284.6 eV as a reference to compensate for the surface charging effects. The coordination state of the single-atomic Zn was characterized by X-ray absorption spectroscopy (XAS). X-ray Absorption Near-Edge Structure (XANES) and Extended X-ray Absorption Fine Structure (EXAFS) analyses in a fluorescence mode were carried out at the 8C beamline of the Pohang Light Source (PLS) in the Pohang Accelerator Laboratory (PAL), Korea. As a reference material for the calibration of the photon energy of each spectrum, a Zn foil was used. ATHENA software was used for the post-processing of XAS data. In processing the data, the Zn K-edge energy from Zn foil was used for the calibration matching to the theoretical value (9659.0 eV). FEFF code and the Artemis software were used for the reduction and data fitting of EXAFS spectra, respectively. NH_3_-temperature programmed desorption (NH_3_-TPD) experiments were carried out using an Automated Catalyst Characterization System (AUTOCHEM II 2920, Micromeritics Instrument Corp.). For the NH_3_-TPD experiment, 100 mg of ZCT SAzyme was pretreated by heating in a flowing stream of argon at 200 °C (10 °C min^-1^) for 30 min in order to remove water and volatile impurities in the samples. After cooling ZCT SAzyme to 50 °C by flowing argon, the flowing gas was switched to NH_3_/He (5 vol%) for 30 min. Then, the reactor was purged with argon and heated from 50 °C to 100 °C (10 °C min^-1^) for 1 hour to remove weakly bound NH_3_. A linear temperature program set to increase from 100 °C to 600 °C at a heating rate of 10 °C min^-1^ was applied for the desorption of NH_3_ that was detected by a thermal conductivity detector (TCD).

**Measurement of catalytic activity: *p*-NPA hydrolysis**

The catalytic activities of ZCT and ZIF-8 were evaluated from chromogenic hydrolysis data of *p*-nitrophenyl acetate (*p*-NPA, 99%, Fluka) to *p*-nitrophenol (*p*-NP). Using a UV–vis spectrophotometer (S-3100, Scinco), the progress of the catalytic hydrolysis was monitored by reading the intensity of a characteristic absorbance peak of *p*-NP at 400 nm. To test the catalytic activity, a *p*-NPA stock solution in acetonitrile was diluted with a phosphate buffer solution to the appropriate concentration (10 mM, pH 7.2). The catalytic reaction was initiated by adding 1 mL of the ZCT SAzyme suspension (2 mg/mL in phosphate buffer solution) to 1 mL of *p*-NPA solution. The initial reaction rate (*V*_0_) was determined from the slope of the *p*-NP production at the early stage of the reaction. The Michaelis-Menten reaction kinetics parameters of Michaelis constant (*K*_m_) and turnover number (*K*_cat_) were determined from the *V*_0_ data obtained at different substrate concentrations. The turnover numbers were calculated based on the Zn content obtained from ICP-OES for each catalyst. To analyze the reusability of ZCT, 1 mg of the SAzymes were dispersed in 1 mL of phosphate buffer solution containing 1 mM of *p*-NPA to proceed the catalytic hydrolysis reaction. After the reaction, ZCT was collected by centrifugation (12,000 rpm, 2 min) and washed with deionized water thrice. The cleaned SAzymes were used for the next cycle of the reaction.

**CO_2_ hydration and sequestration**

To test the ZCT for practical applicability for CO_2_ conversion, CO_2_-saturated deionized water was prepared by bubbling CO_2_ gas into deionized water in a round-bottomed flask under ice-cold conditions. To the 10 mL ZCT dispersion (1 mg/mL) in Tris-HCl buffer solution (10 mM, pH 8.5), 3 mL of CO_2_-saturated water was added to induce CO_2_ hydration. Progress of CO_2_ hydration was traced by monitoring the pH change over time. Experiments for CO_2_ sequestration to CaCO_3_ were performed by the addition of 10 mM of CaCl_2_ solution to the hydrated CO_2_ solution. The weights of the CaCO_3_ precipitates were measured after drying them in a vacuum oven at 100 °C to remove water.

**DFT calculation**

Vienna *Ab Initio* Simulation Package (VASP) was utilized to understand the CO_2_ conversion reaction on the catalyst.^[S1,S2]^ We employed projected augmented wave (PAW) potentials^[S3]^ and the Perdew-Burke-Ernzerhof (PBE) exchange-correlation functional^[S4]^ with the D3-Grimme method,^[S5]^ which accounts for the van der Waals correction. The criteria for energy and force convergence were set at 10^-5^ eV and 0.02 eV/Å, respectively. A 5 × 1 × 1 *K*-point mesh was applied to the model systems. The adsorption energy of OH- is calculated using the equation *E*(OH^-^*) - *E*(*) - *E*(OH^-^), where *E*(OH^-^*), *E*(*), and *E*(OH^-^) represent the energies of the OH^-^ adsorbed systems, the pristine model systems, and the free OH^-^ molecule, respectively. The energy of OH^-^ is determined by the formula *E*(H_2_O) - 0.5*E*(H_2_) - *RT*ln[H^+^], where *E*(X) represents the energy of molecule X, *R* the gas constant, and *T* room temperature, assuming that H_2_ (g) and H^+^ (aq) at pH 1 are in equilibrium. The adsorption energies depending on the diheral angle align with experimental results of the catalytic activity when the Zn–N bond length is set to 2.19 Å, whereas deviations are observed when the bond length is closer to the experimentally measured value of 2.035 Å. To elucidate the effect of the dihedral angle on catalytic activity, we fixed the Zn–N bond length at 2.19 Å in our calculations. The transition state energy barriers were calculated using the climbing image nudged elastic band (c-NEB) method,^[S6]^ with a convergence criterion of 0.07 eV/Å. The integrated Crystal Orbital Hamilton Population (iCOHP) was calculated by local-orbital basis suite towards electronic structure reconstruction (LOBSTER).^[S7,S8]^ Crystal Orbital Hamilton Population (COHP), is a computational technique used to analyze the bonding interactions within a material by examining the contributions of specific atomic orbitals to the electronic structure. It’s particularly useful in solid-state chemistry and materials science to understand how atoms within a crystal lattice interact.


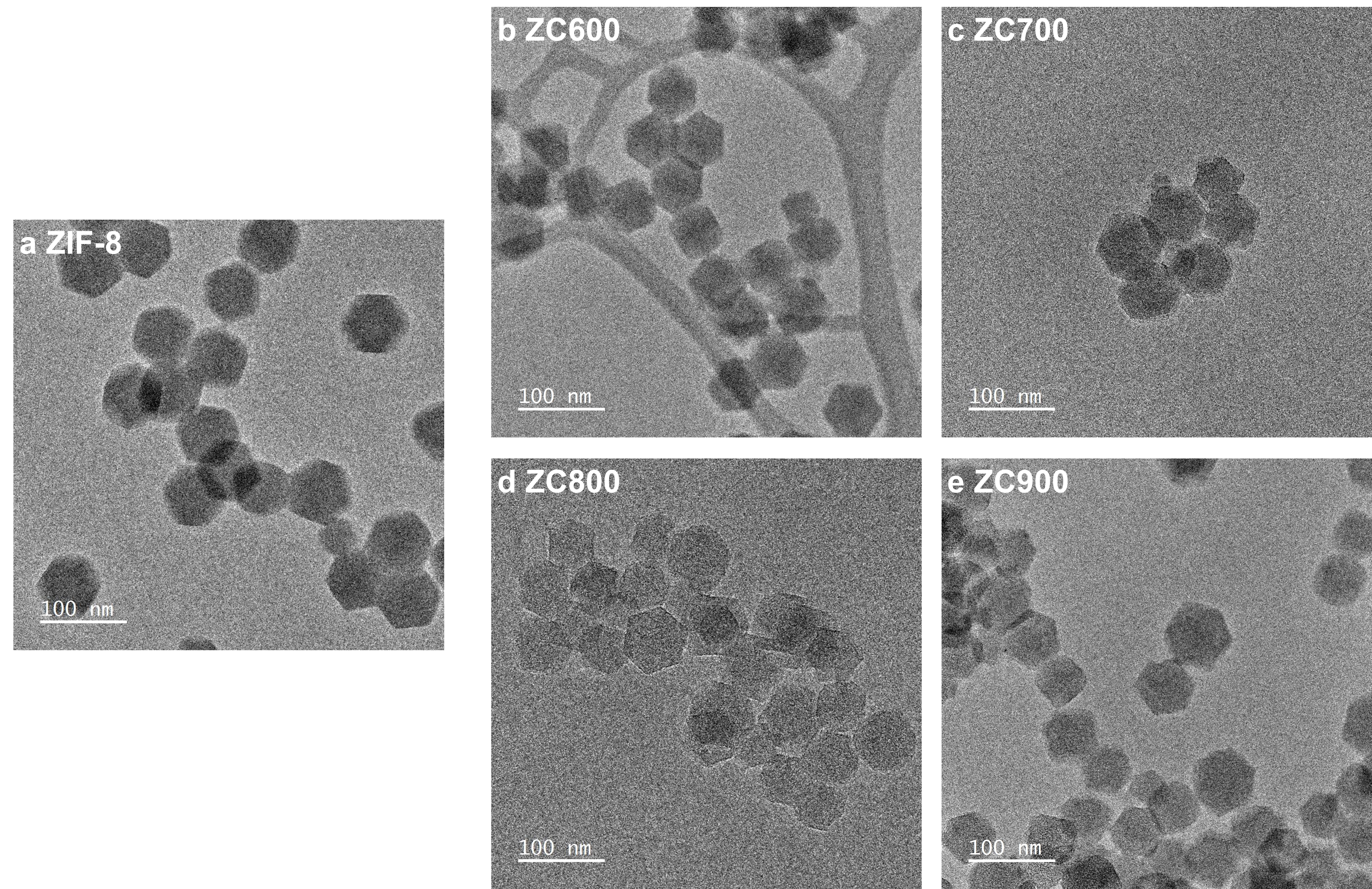


**Supplementary Figure S1.** TEM images of **(a)** ZIF-8 and **(b-e)** ZCT SAzymes.


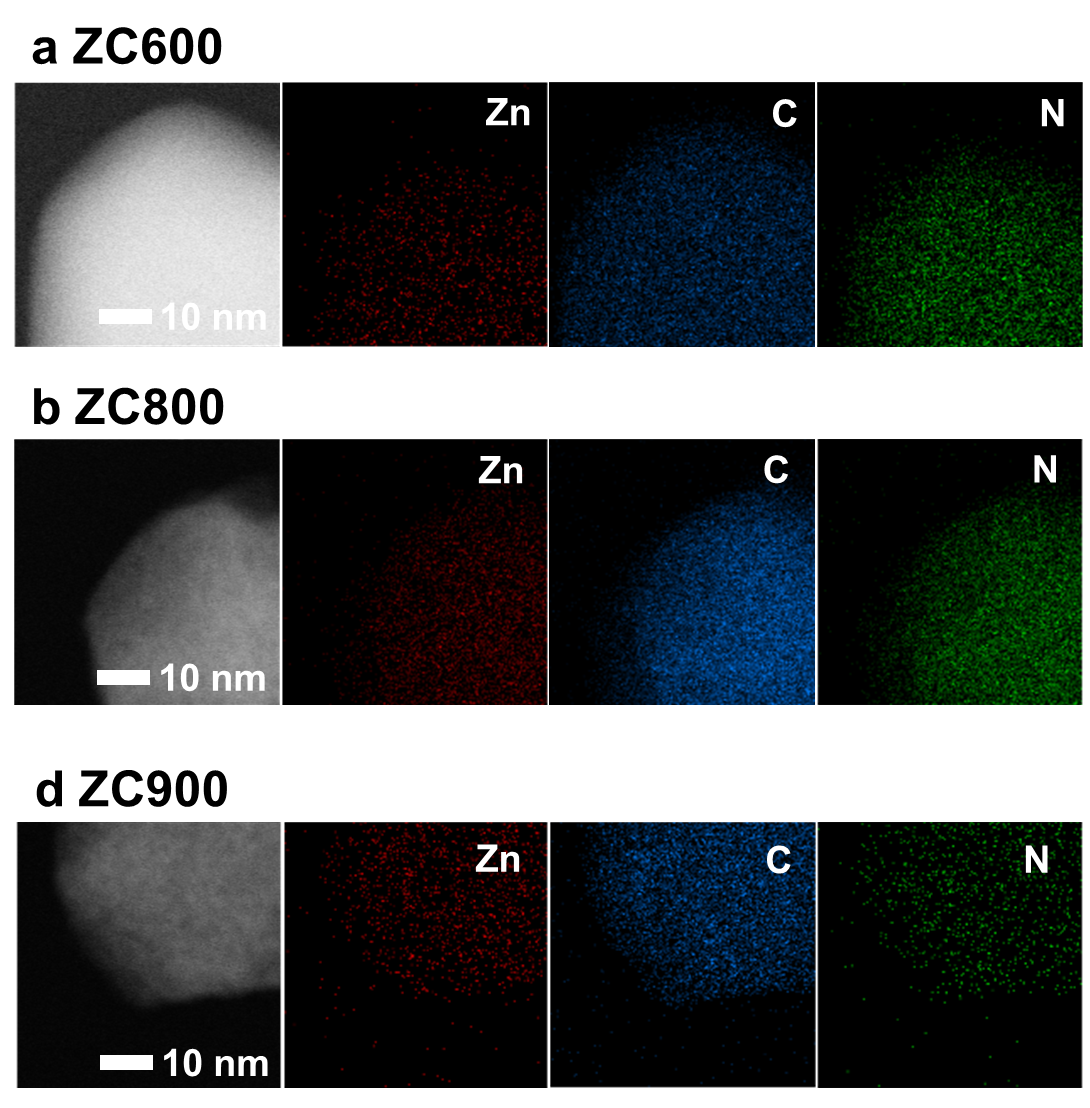


**Supplementary Figure S2.** TEM and elemental mapping images of **(a)** ZC600, **(b)** ZC800, and **(c)** ZC900.

**Supplementary Figure S3.** XRD patterns of as-carbonized ZCTs without acid-washing.


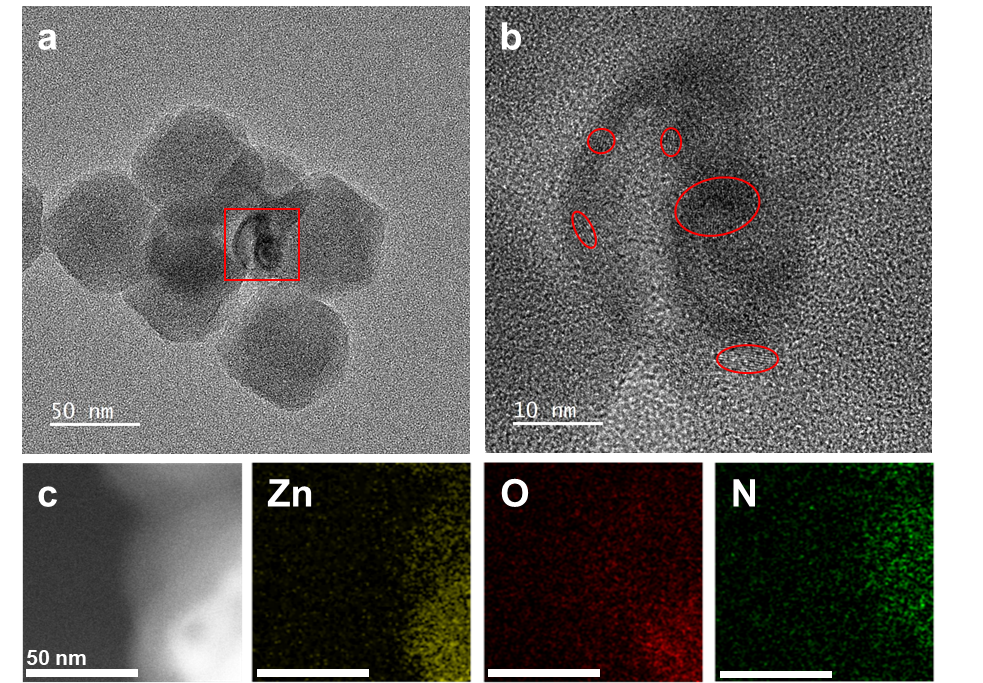


**Supplementary Figure S4.** **(a, b)** TEM images and **(c)** elemental mapping of as-carbonized ZC700 before acid-washing. **(b)** Image of the red rectangular region in **(a)** in higher magnitude. Crystalline domains are marked with red circles.

**Supplementary Figure S5.** Thermogravimetric analysis (TGA) of ZIF-8 under N_2_.

**
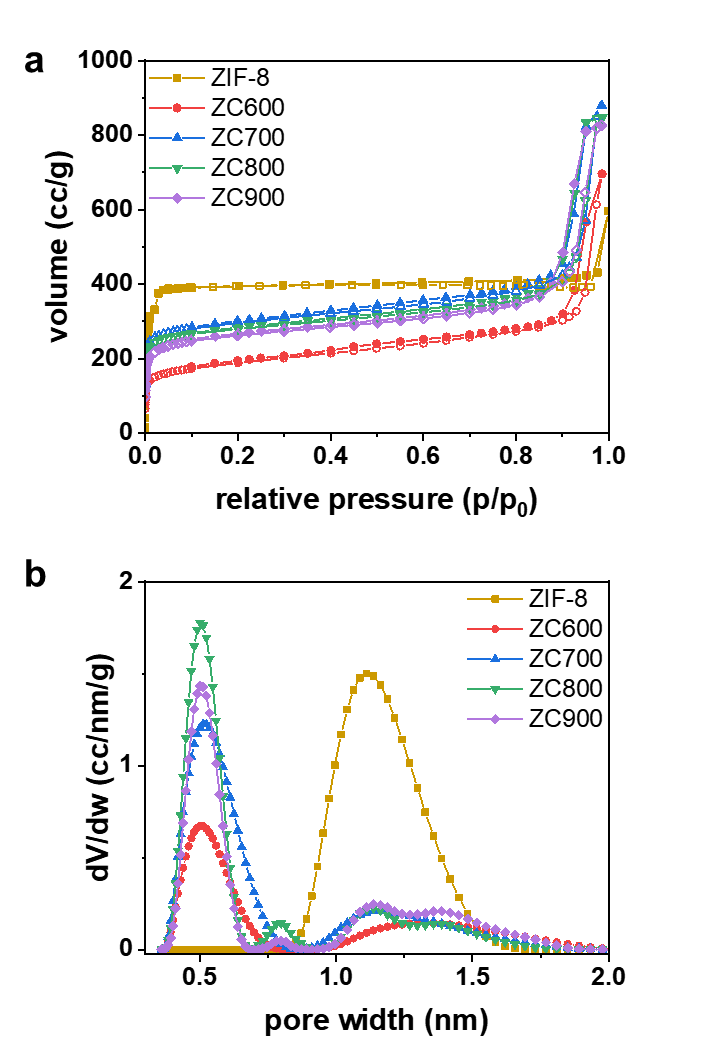
**

**Supplementary Figure S6. (a)** N_2_ isotherms at 77 K and **(b)** pore size distribution of ZIF-8 and ZCT SAzymes.

**Supplementary Table S1.** Summary of BET analysis of ZIF-8 and ZCT SAzymes.

|  | **BET surface area**  **(m2/g)** | **Micropore volume**  **(cc/g)** |
| --- | --- | --- |
| ZC600 | 691 | 0.17 |
| ZC700 | 1126 | 0.34 |
| ZC800 | 1072 | 0.33 |
| ZC900 | 993 | 0.30 |
| ZIF-8 | 1716 | 0.59 |


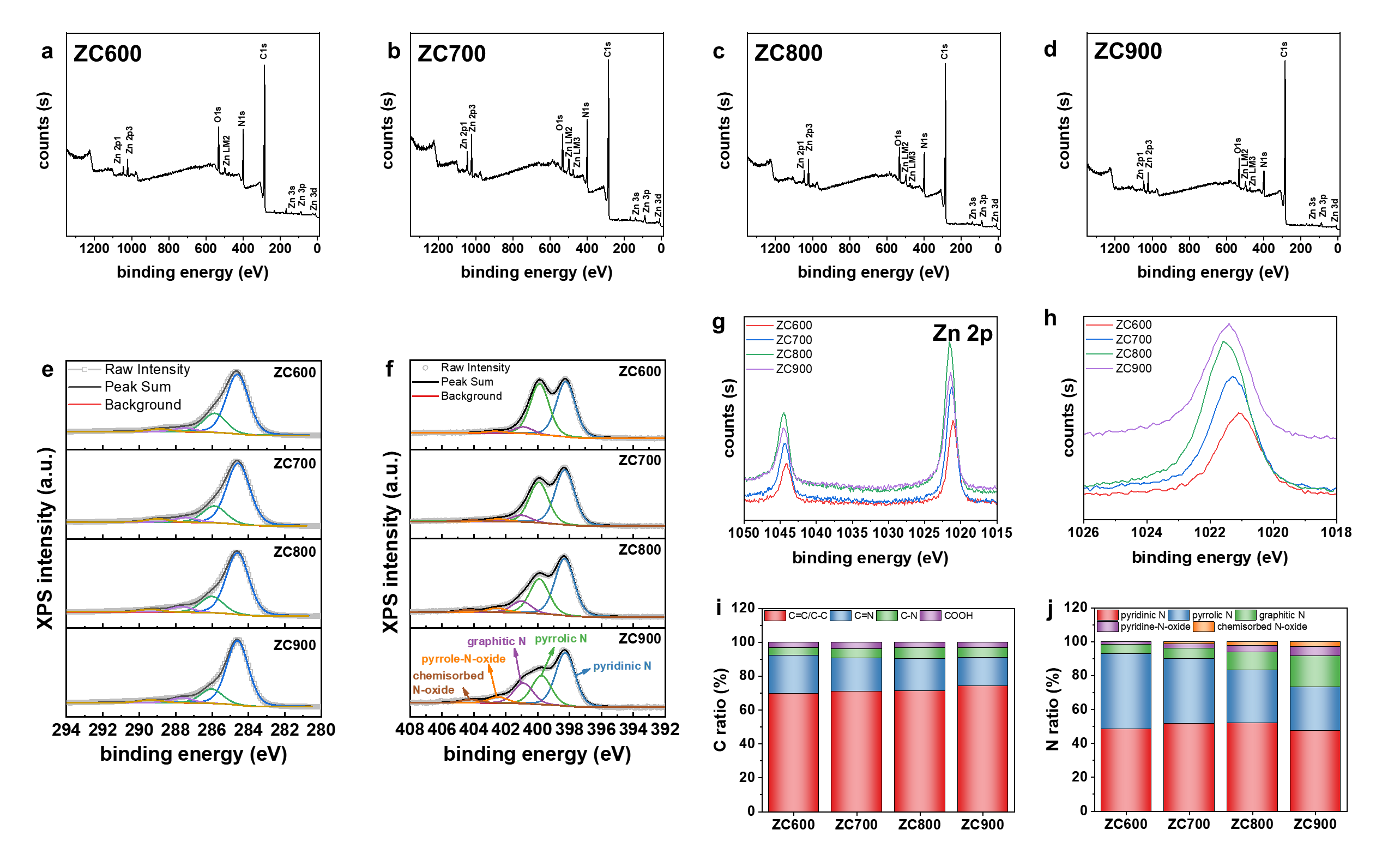


**Supplementary Figure S7.** XPS analysis of ZCT SAzymes. **(a-d)** Survey spectra, **(e-h)** high-resolution C1s, N1s, Zn2p, and enlarged Zn2p_3/2_ spectra, respectively, **(i, j)** contents of carbon and nitrogen compounds at various carbonization temperatures.

**Supplementary Table S2.** Atomic contents of ZCT SAzymes determined by XPS.

| **Sample** | **Components (at%)** | | | |
| --- | --- | --- | --- | --- |
|  | C | N | O | Zn |
| ZC600 | 71.8 | 20.2 | 7.7 | 0.3 |
| ZC700 | 76.8 | 16.4 | 6.4 | 0.4 |
| ZC800 | 78.7 | 15.5 | 5.2 | 0.6 |
| ZC900 | 83.9 | 10.3 | 5.4 | 0.4 |

**Supplementary Figure S8.** Raman spectra of ZCT SAzymes.

**Supplementary Figure S9.** FT-IR spectra of ZIF-8 and ZCTs.

**
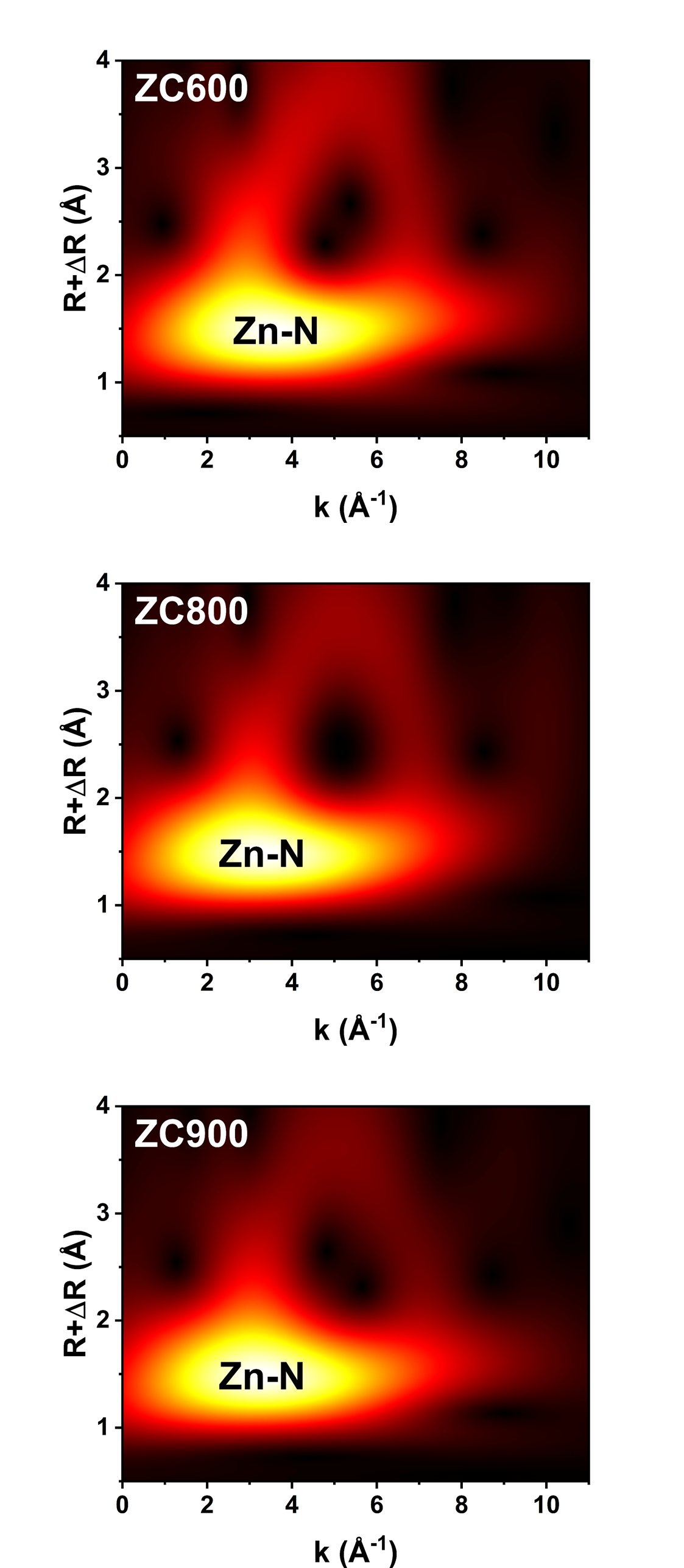
**

**Supplementary Figure S10.** Wavelet Transformed-EXAFS contour plots of ZCT SAzymes.

**Supplementary Figure S11.** XANES spectra of Zn references and ZCT SAzymes.

**Supplementary Figure S12.** Full profiles of NH_3_-TPD analyses of ZCT SAzymes.


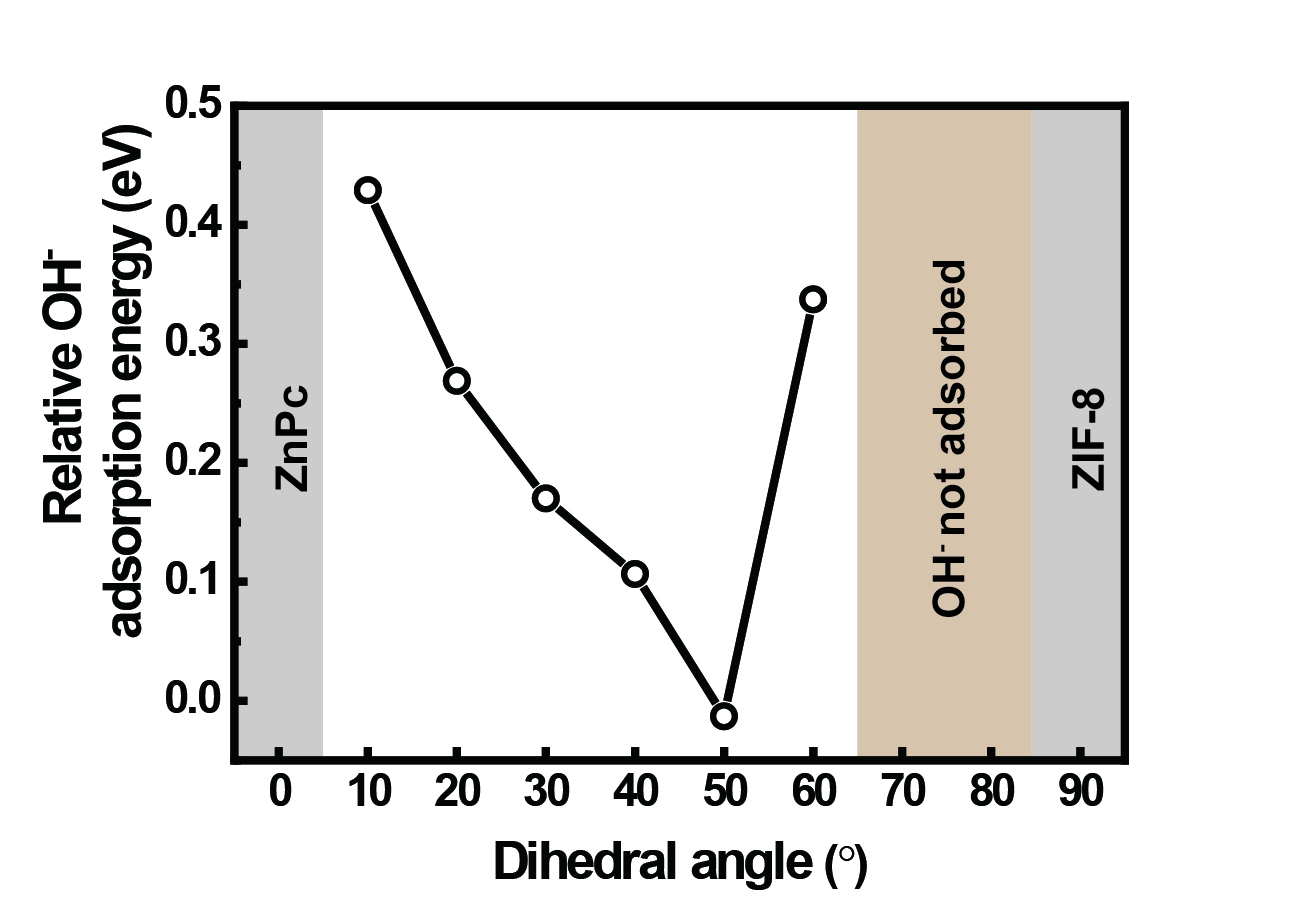


**Supplementary Figure S13.** The relative OH^-^ adsorption energy as a function of dihedral angles compared to the dihedral angle of 50°. In this calculation, we excluded the 90° and 0° dihedral angles because the coordination geometries of ZCT SAzymes exhibit a Zn-XANES spectral profile, shown in Figure 3a, that is distinctly different from those of ZIF-8 and ZnPc, which have tetrahedral or square planar structures. Additionally, dihedral angles exceeding 60° were excluded, as OH⁻ does not adsorb at these angles.

**Supplementary Figure S14.** OH⁻ adsorption energy as a function of Zn-N bond length and dihedral angle.

**
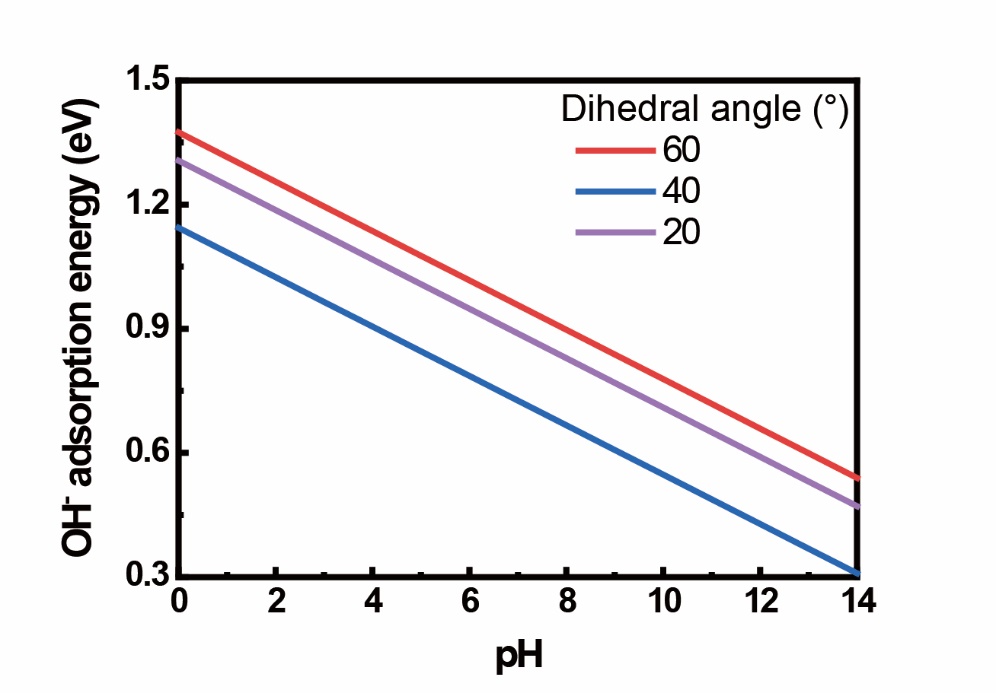
**

**Supplementary Figure S15.** OH^-^ adsorption energy as a function of pH for Zn–N_4_ geometries in dihedral angles of 60°, 40°, and 20°


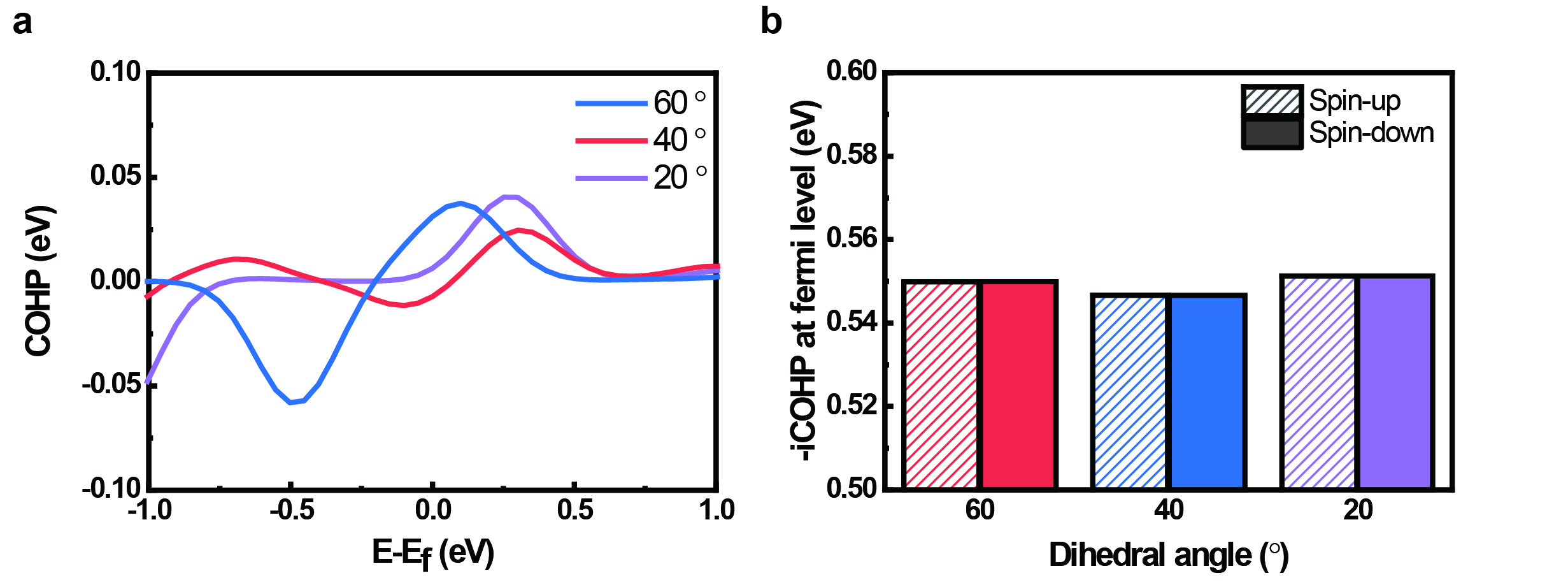


**Supplementary Figure S16. (a)** COHP between Zn and O in OH adsorbed molecule according to the dihedral angles of 20, 40 and 60 degrees. **(b)** The averaged -iCOHP at Fermi level as a function of dihedral angles for spin up and down of Zn-N bonds.


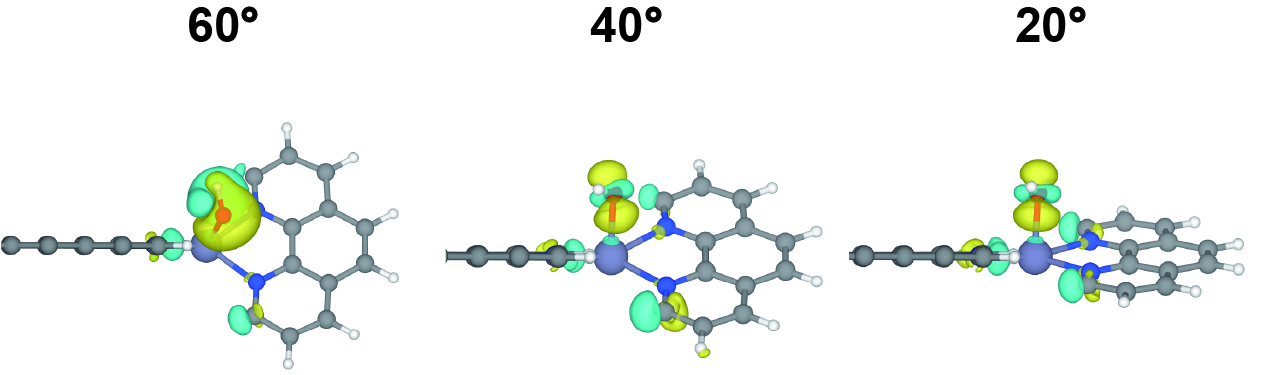


**Supplementary Figure S17.** charge density distribution of ZCT SAzymes.


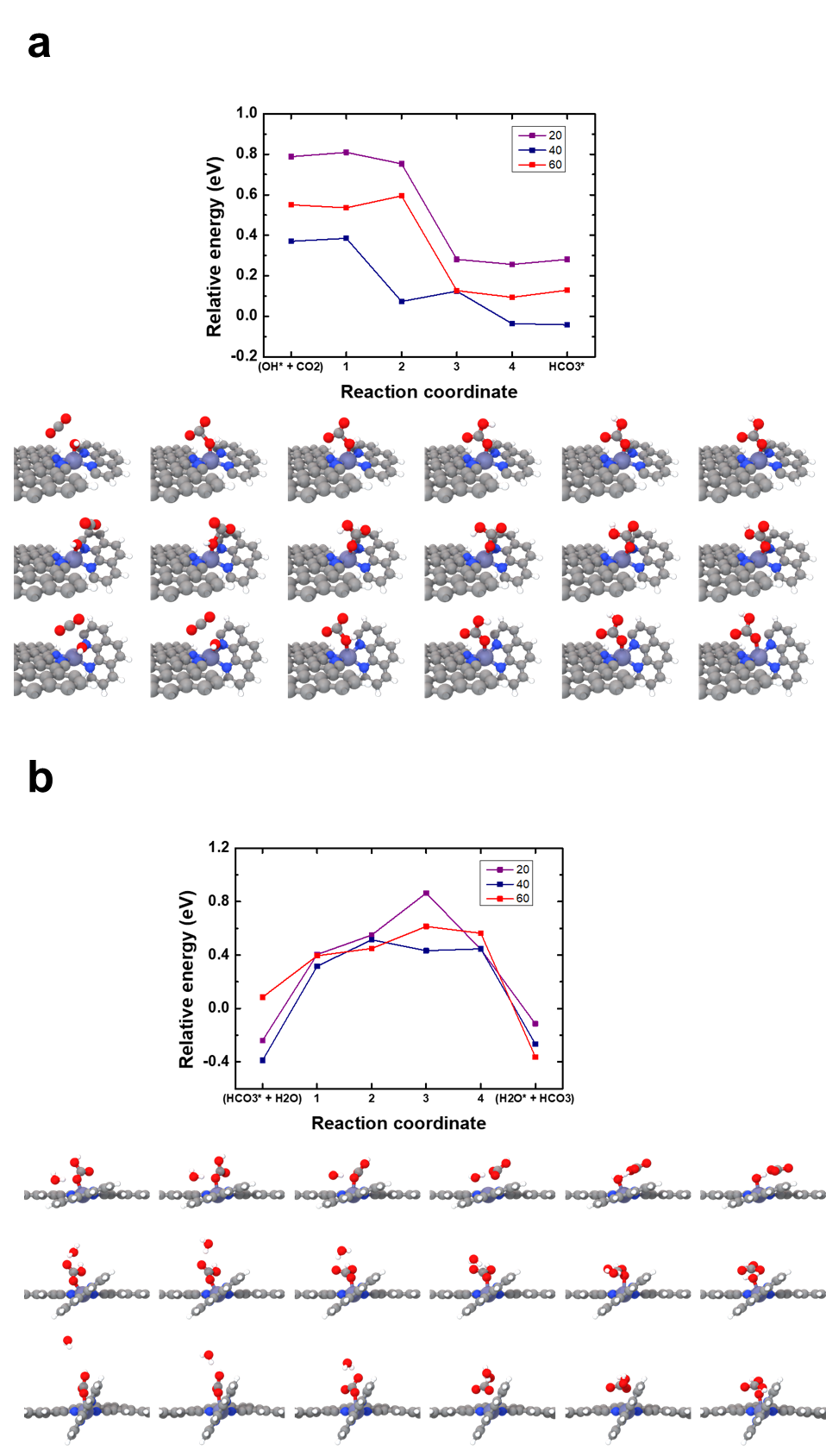


**Supplementary Figure S18. Reaction diagrams of (a)** HCO_3_^-^ formation from free CO_2_ molecule and OH^-^ on Zn-N_4_ sites and **(b)** substitution of HCO_3_* with H_2_O on Zn-N_4_ active sites of 20 degree, 40 degree and 60 degree model systems, generating HCO_3_^-^. The illustrations in first, second and third rows below the plots present the snapshots of the reactions on the ZnN_4_ active sites with tilted angles of 20 degree, 40 degree and 60 degree, respectively. The activation energies in (a) and (b) are determined by the difference between the highest energy attained during the reaction and the initial state energy. Reactions are presumed to occur under charge conservation enviornments.

**
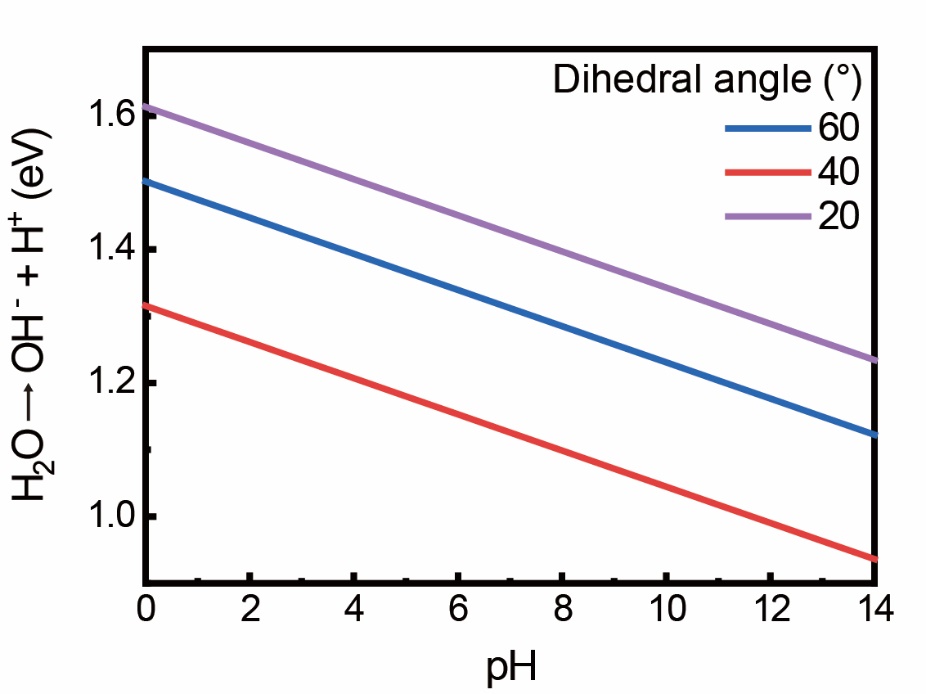
**

**Supplementary Figure S19.** Deprotonation energy as a function of pH for the Zn–N_4_ geometries in dihedral angles of 60°, 40°, and 20°

**
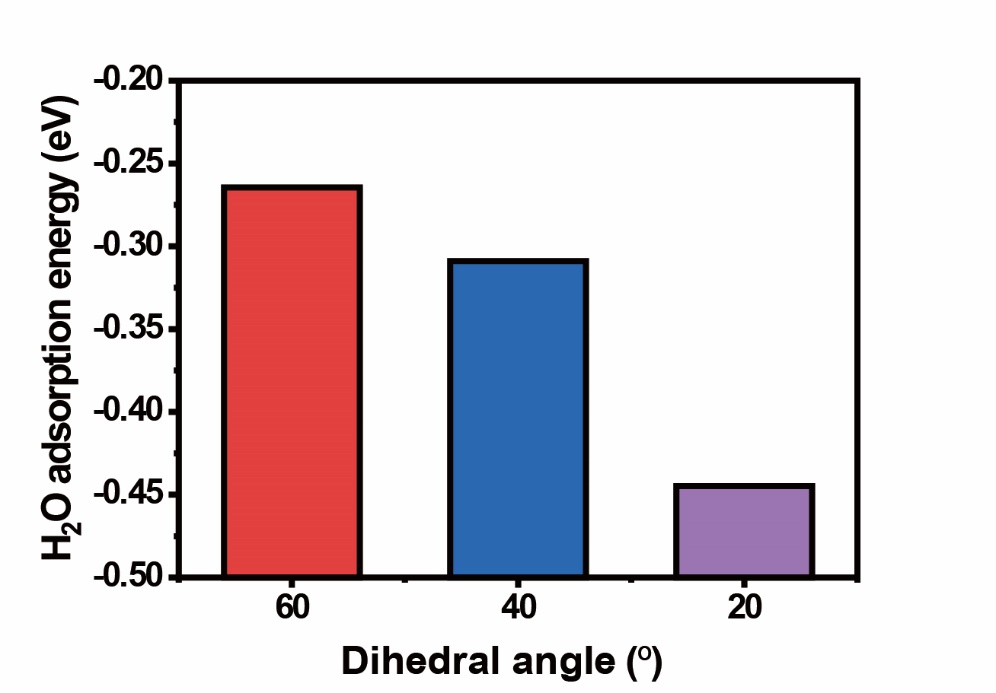
**

**Supplementary Figure S20.** Adsorption energy of H_2_O as a function of pH for the Zn–N_4_ geometries in dihedral angles of 60°, 40°, and 20°.

**
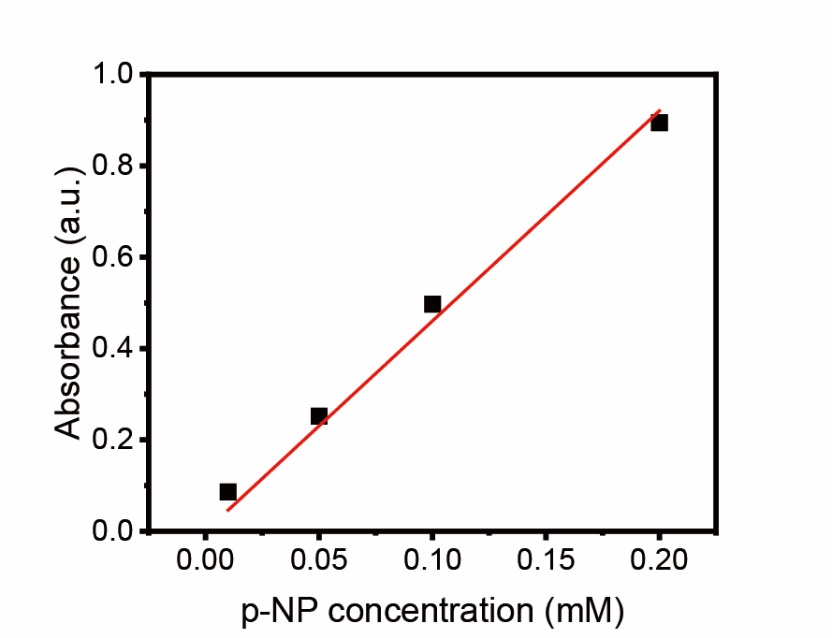
**

**Supplementary Figure S21.** Standard calibration curve of *p*-NP.

**
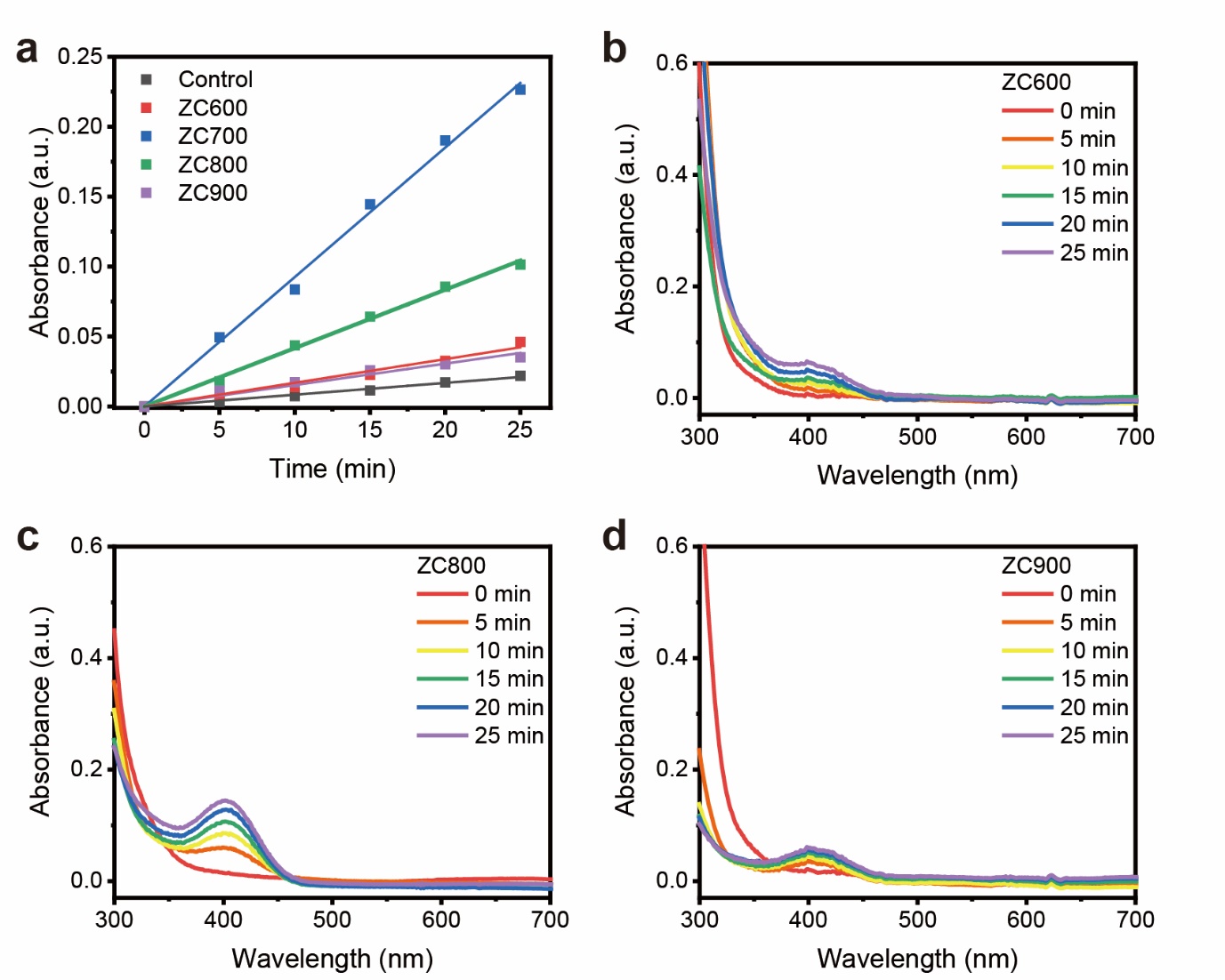
**

**Supplementary Figure S22. (a)** Increase of absorbance intensity of *p*-NP (at λ = 400 nm) with the progress of the catalytic *p*-NPA hydrolysis by ZCT SAzymes. Control represents the absorbance changes in the absence of ZCT catalysts by the natural hydrolysis of *p*-NPA. **(b-d)** UV-Vis absorbance spectra of *p*-NPA solution in the presence of ZC600, ZC800, and ZC900 SAzymes over time.

**
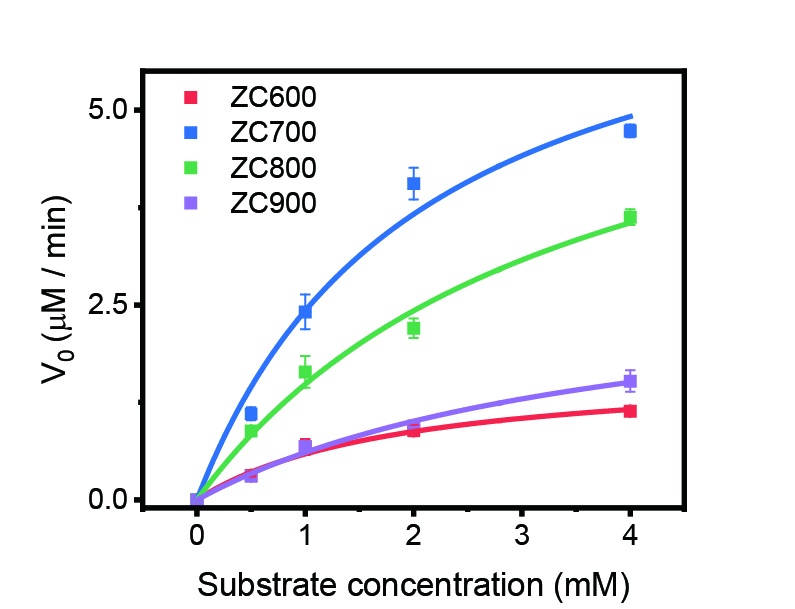
**

**Supplementary Figure S23.** Initial reaction rate (*V*_0_) of *p*-NPA hydrolysis at various substrate concentrations in the presence of ZCT SAzymes. Lines were fitted curves by Michaelis-Menten equation.

**Supplementary Table S3. Michaelis-Menten kinetics parameters for *p*-NPA hydrolysis**

|  | ***k*_cat_** | ***K*_m_** | ***k*_cat_/*K*_m_** | **(*k*_cat_/*K*_m_)/SA** |
| --- | --- | --- | --- | --- |
|  | **(min^-1^)** | **(M)** | **(M^-1^ min^-1^)** | **(M^-1^min^-1^ m^-2^ mg)** |
| **ZIF-8** | 0.034 | 0.012 | 2.78 | **1.62** |
| **ZC600** | 0.0023 | 0.0019 | 1.19 | **1.72** |
| **ZC700** | 0.0091 | 0.0021 | 4.34 | **3.86** |
| **ZC800** | 0.0062 | 0.0035 | 1.79 | **1.67** |
| **ZC900** | 0.0033 | 0.0039 | 0.83 | **0.84** |
| **ZnPc**^a^ | 0.0042 | 0.0053 | 0.80 | - |

^a^*p*-NPA hydrolysis was conducted using dimethyl sulfoxide as a co-solvent to dissolve ZnPc.


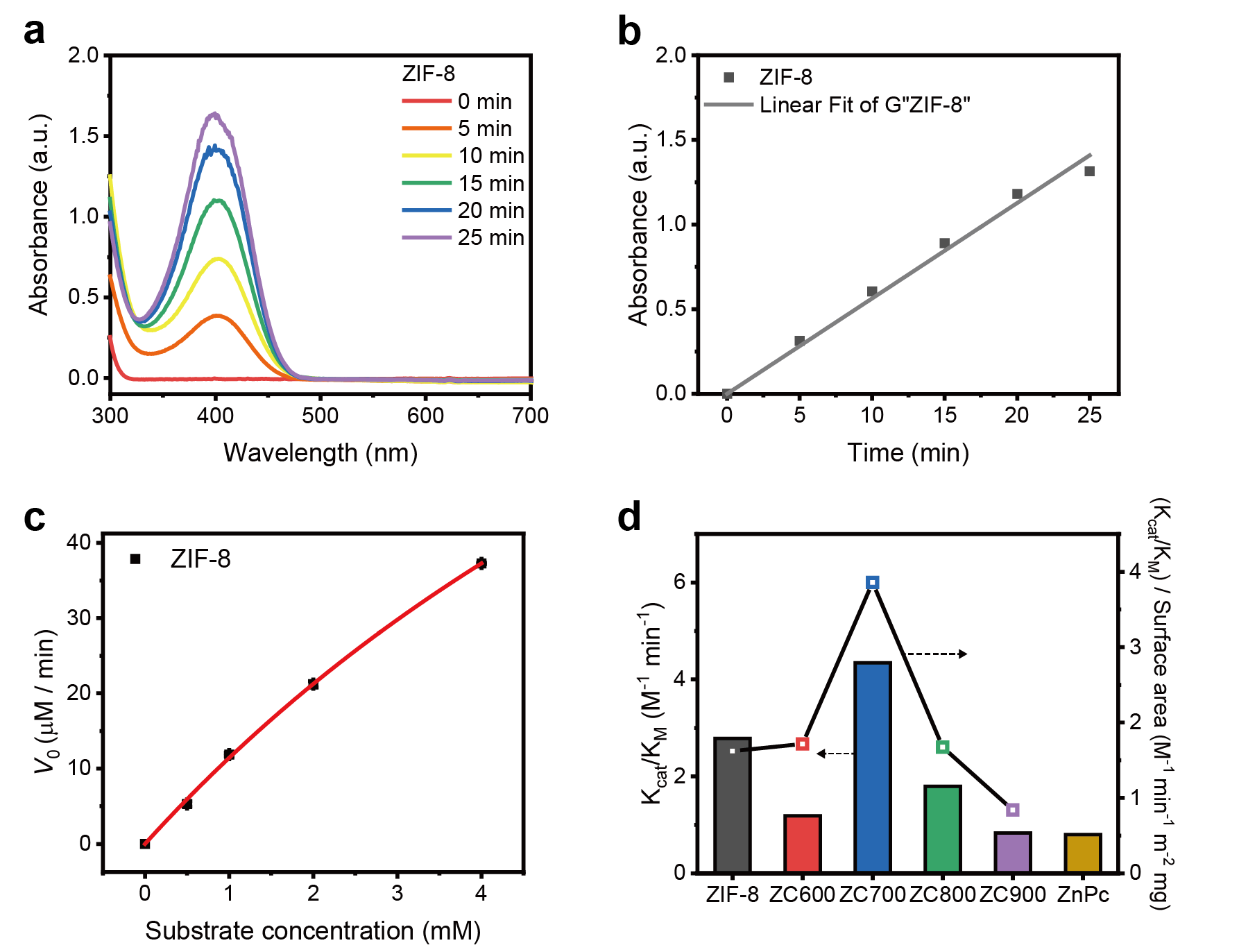


**Supplementary Figure S24. (a)** UV-Visible absorbance spectra of *p*-NPA solution in the presence of ZIF-8 (1 mg/mL) over time. **(b)** Increase of absorbance intensity of *p*-NP (at 400 nm) with progress of the catalytic *p*-NPA hydrolysis by ZIF-8. **(c)** The initial reaction rate (*V*_0_) of *p*-NPA hydrolysis at various substrate concentrations in the presence of ZIF-8. The line was fitted by Michaelis-Menten equation. **(d)** Comparison of catalytic efficiency (*K*_cat_/*K*_m_) and surface area-normalized efficiency (*K*_cat_/*K*_m_ ∙ surface area) of ZIF-8 and ZnPc with ZCT SAZymes. Catalytic activity was evaluated based on the Zn content determined by ICP-OES for each sample.

**
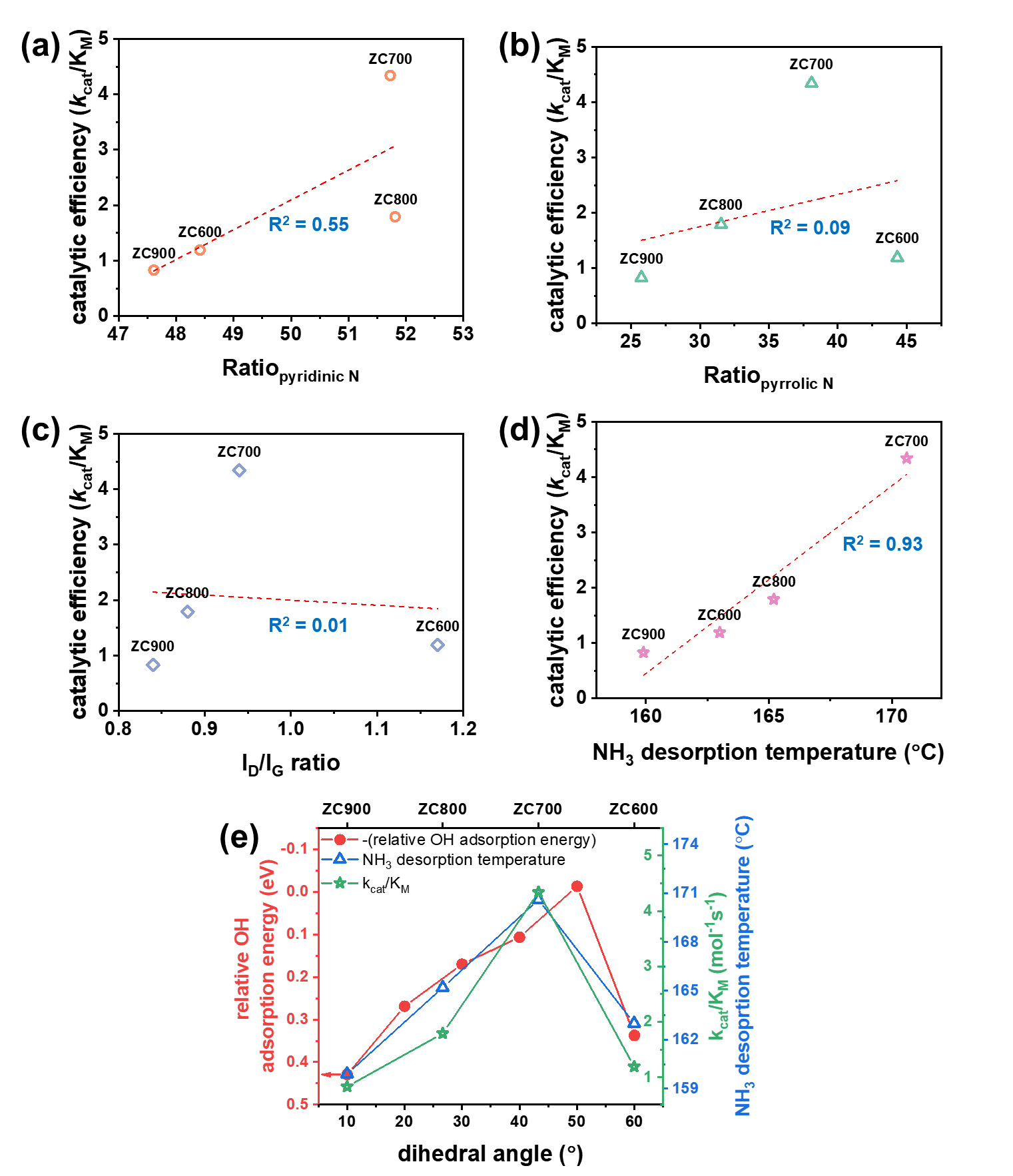
**

**Supplementary Figure S25.** Correlation between the catalytic efficiency (k_cat_/K_M_) and the ratio of **(a)** pyridinic N, **(b)** pyrrolic N, **(c)** I_D_/I_G_, **(d)** and NH_3_ desorption temperature. **(e)** Comparison of the DFT calculated dihedral angle vs. relative OH^-^ adsorption energy graph and ZCT SAzyme vs. k_cat_/K_M_ and NH_3_ desorption temperature graphs.


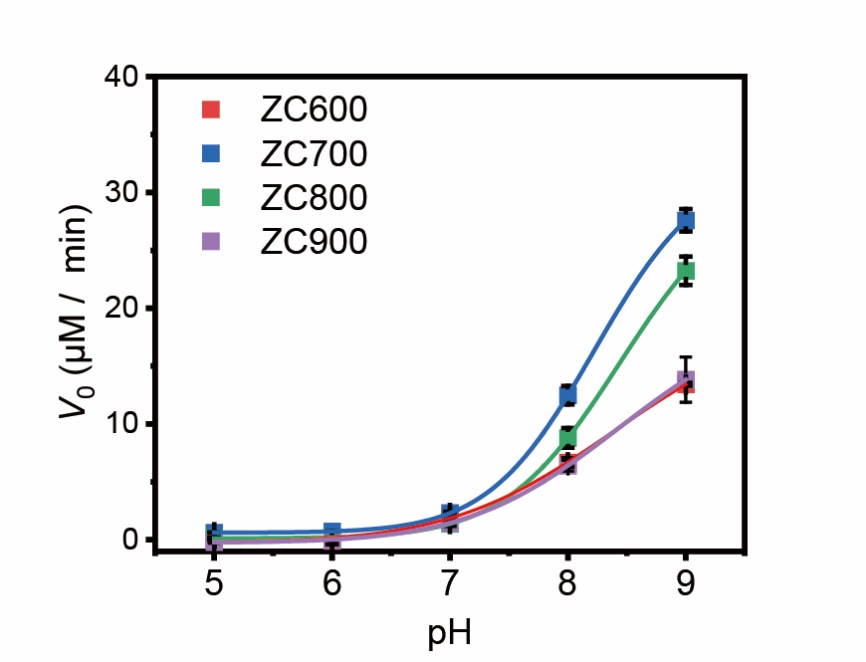


**Supplementary Figure S26.** Variation of initial hydrolysis rate (*V*_0_) of *p*-NPA at different pHs.

Supplementary Table S4. Catalytic efficiencies and parameters of bovine carbonic anhydrase (BCA) and nanozymes.

| **Catalyst** | **Supports** | **pH** | **E_a_** | ***V_max_*** | ***k*_cat_** | ***K*_m_** | ***k*_cat_/*K*_m_** | **Ref** |
| --- | --- | --- | --- | --- | --- | --- | --- | --- |
|  |  |  | **(kJ mol^-1^)** | **(nM s^-1^)** | **(10^-3^ s^-1^)** | **(mM)** | **(M^-1^ s^-1^)** |  |
| **BCA** | - | 8.0 | - | - | 49.51×10^-3^ | 56.67 | 873.76 | [S9] |
| **BCA** | - | 7.5 | 4.69 | - | - | - | - | [S10] |
| **His-C7[Zn]** | Peptide self-assembly | 7.0 | 29.7 | - | 457 | 164.8 | 2.77 | [S11] |
| **VFFAHH assembly** | Peptide self-assembly | 7.4 | 44 | - | 1.07 | 0.63 | 1.7 | [S12] |
| **CMNT** | LbL nanotubes | 6.5 | 17.9 | - | 62.3 | 33.57 | 1.85 | [S13] |
| **ZIF-8** | MOF | 7.2 | - | 2.53 | 0.567 | 12 | 0.046 | This Work |
| **ZC700** | Carbon | 7.2 | 19.4 | 0.13 | 0.152 | 2.1 | 0.072 |


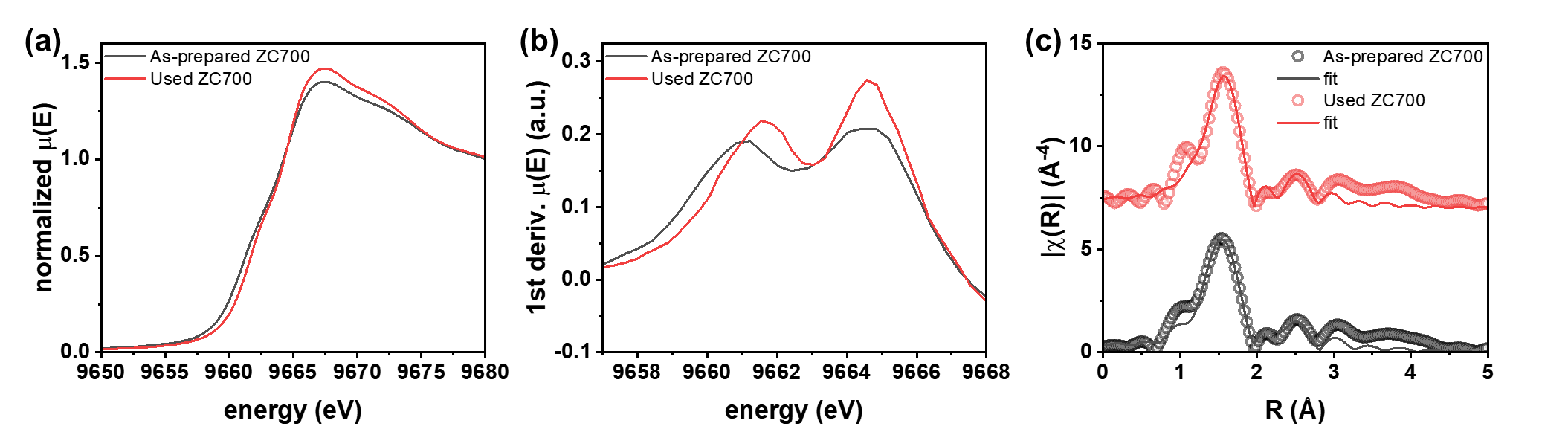


**Supplementary Figure S27.** (a) Normalized XANES, (b) 1^st^ derivative of XANES, and (c) FT-EXAFS spectrum and its best fit of the used ZC700. XANES and EXAFS spectra of the as-prepared ZC700 are the same data shown in Figure 2(d), 3(a), and Supplementary Figure 11.

Supplementary Table S5. FT-EXAFS fitting result of the used ZC700.

|  | **Path** | **N^a^** | **Distance (Å)** | **Debye-Waller factor (Å^2^)** | **R-factor^b^** |
| --- | --- | --- | --- | --- | --- |
| **used ZC700** | Zn–N/O | 5.49(0.42) | 2.045(0.007) | 0.009 (0.001) | 0.009 |
|  | Zn–C | 2.17(0.46) | 3.023(0.020) | 0.002 (0.001) |  |


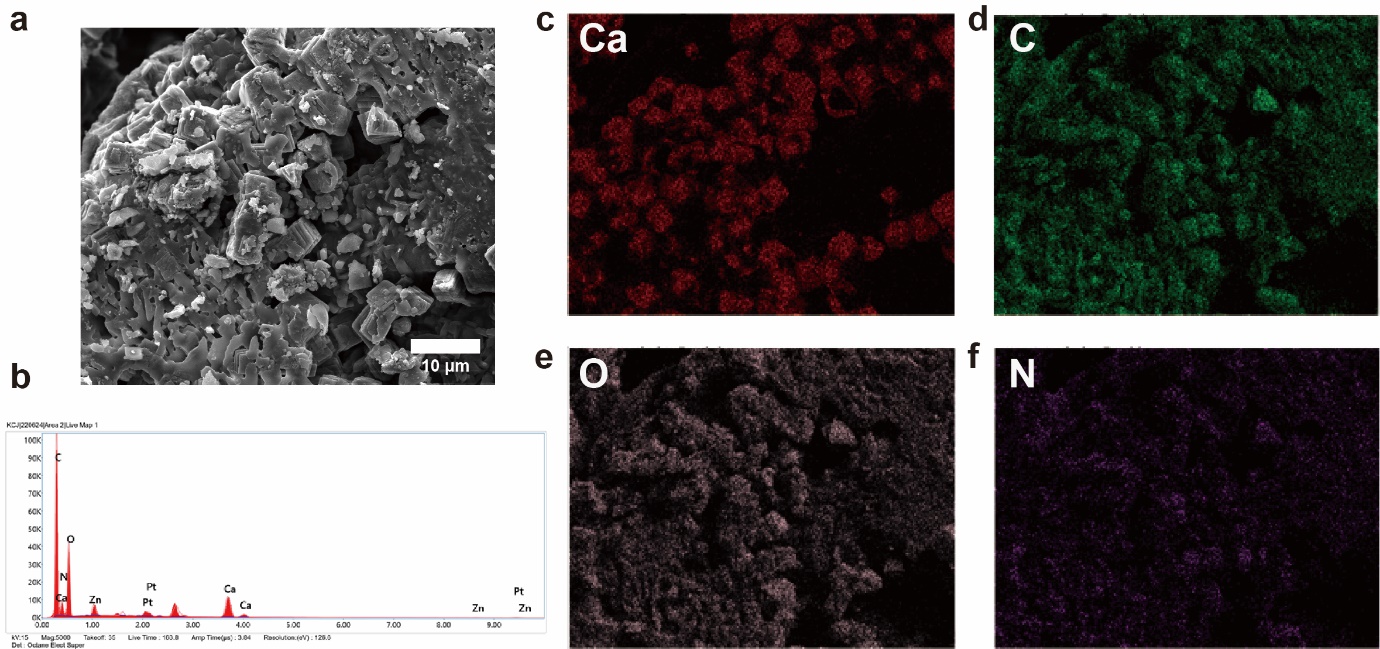


**Supplementary Figure S28. (a)** Scanning electron micrograph (SEM) image of the CaCO_3_ precipitates obtained from the mixture of catalytically hydrolyzed CO_2_ solution and CaCl_2_. **(b-f)** Energy dispersive X-ray spectrum of the precipitate and atomic mapping images showing calcium (Ca), carbon (C), oxygen (O), and nitrogen (N).

**References**

[S1] G. Kresse, J. Hafner, *Phys. Rev. B* **1993**, *47*, 558–561.

[S2] G. Kresse, J. Furthmüller, *Comput. Mater. Sci.* **1996**, *6*, 15–50.

[S3] P. E. Blöchl, *Phys. Rev. B* **1994**, *50*, 17953–17979.

[S4] K. Li, L. Luo, Y. Zhang, W. Li, Y. Hou, *ACS Appl. Mater. Interfaces* **2018**, *10*, 41525–41534.

[S5] S. Grimme, J. Antony, S. Ehrlich, H. Krieg, *J. Chem. Phys.* **2010**, *132*, DOI 10.1063/1.3382344.

[S6] H. JÓNSSON, G. MILLS, K. W. JACOBSEN, in *Class. Quantum Dyn. Condens. Phase Simulations*, WORLD SCIENTIFIC, **1998**, pp. 385–404.

[S7] R. Dronskowski, P. E. Bloechl, *J. Phys. Chem.* **1993**, *97*, 8617–8624.

[S8] V. L. Deringer, A. L. Tchougréeff, R. Dronskowski, *J. Phys. Chem. A* **2011**, *115*, 5461–5466.

[S9] M. Vinoba, D. H. Kim, K. S. Lim, S. K. Jeong, S. W. Lee, M. Alagar, *Energy & Fuels* **2011**, *25*, 438–445.

[S10] N. Sarraf, A. Saboury, B. Ranjbar, A. Moosavi-Movahedi, *Acta Biochim. Pol.* **2004**, *51*, 665–671.

[S11] M.-C. Kim, S.-Y. Lee, *ChemCatChem* **2015**, *7*, 698–704.

[S12] A. Singh, J. P. Joseph, D. Gupta, C. Miglani, N. A. Mavlankar, A. Pal, *Nanoscale* **2021**, *13*, 13401–13409.

[S13] M. Kim, H. Lee, Y. Kim, M.-C. Kim, S.-Y. Lee, *ACS Sustain. Chem. Eng.* **2024**, *12*, 13415–13426.
